# Introgression of a functional epigenetic *OsSPL*1*4*^WFP^ allele into elite indica rice genomes greatly improved panicle traits and grain yield

**DOI:** 10.1038/s41598-018-21355-4

**Published:** 2018-03-01

**Authors:** Sung-Ryul Kim, Joie M. Ramos, Rona Joy M. Hizon, Motoyuki Ashikari, Parminder S. Virk, Edgar A. Torres, Eero Nissila, Kshirod K. Jena

**Affiliations:** 1 Strategic Innovation Platform, International Rice Research Institute (IRRI), DAPO Box, 7777 Metro Manila, Philippines; 2 Bioscience and Biotechnology Center, Nagoya University, Nagoya, Japan; 3 International Center for Tropical Agriculture (CIAT), A.A, 6713 Cali, Colombia; 4 Rice Tech LTDA, Santa Maria, RS Brazil

## Abstract

Rice yield potential has been stagnant since the Green Revolution in the late 1960s, especially in tropical rice cultivars. We evaluated the effect of two major genes that regulate grain number, *Gn1a/OsCKX2* and *IPA1/WFP/OsSPL14*, in elite indica cultivar backgrounds. The yield-positive *Gn1a*-type 3 and *OsSPL14*^WFP^ alleles were introgressed respectively through marker-assisted selection (MAS). The grain numbers per panicle (GNPP) were compared between the recipient allele and the donor allele groups using segregating plants in BC_3_F_2_ and BC_3_F_3_ generations. There was no significant difference in GNPP between the two *Gn1a* alleles, suggesting that the *Gn1a*-type 3 allele was not effective in indica cultivars. However, the *OsSPL14*^WFP^ allele dramatically increased GNPP by 10.6–59.3% in all four different backgrounds across cropping seasons and generations, indicating that this allele provides strong genetic gain to elite indica cultivars. Eventually, five high-yielding breeding lines were bred using the *OsSPL14*^WFP^ allele by MAS with a conventional breeding approach that showed increased grain yield by 28.4–83.5% (7.87–12.89 t/ha) vis-à-vis the recipient cultivars and exhibited higher yield (~64.7%) than the top-yielding check cultivar, IRRI 156 (7.82 t/ha). We demonstrated a strong possibility to increase the genetic yield potential of indica rice varieties through allele mining and its application.

## Introduction

Rice (*Oryza sativa* L.) is one of the most important cereal crops and it feeds more than half of the world’s population. People in most Asian countries have been eating rice as a staple food, and rice consumption is rapidly growing in African countries^1^. The human population is rising continuously and urbanization/industrialization is reducing the area of crop land and climate changes is threatening stable crop production. In contrast, the rates of global yield increase of the major crops are insufficient to meet food demand for the estimated nine billion people by 2050^2,3^. Significant improvement of genetic yield potential per unit area is one of the most important goals in rice genetic research and breeding programs.

Rice grain productivity is a complex trait. It is directly or indirectly influenced by many other traits, such as plant architecture, heading date, and the degree of resistance to biotic/abiotic stresses. Four major traits, grain size, grain filling ability, grain numbers per panicle (GNPP), and tiller number per plant, are directly associated with rice grain productivity. However, these traits are complex and quantitative in nature. Quantitative trait loci (QTL) analysis with fine mapping and positional cloning from rice mutants identified about 20 genes in rice that controlled these traits^4–9^. Although around 20 yield-related genes have been identified in rice, strangely, the evaluation and use of the yield-positive alleles are still very limited in rice research activities as well as in actual rice breeding programs. Only a few alleles of yield-related genes have been tested in early generations or in japonica variety backgrounds. The effects of the *dep1* allele producing dense and erect panicles^10^ and the *OsSPL14*^IPA^ allele providing ideal plant architecture^11^ were tested in two F_2_ populations^12^. The near-isogenic lines (NILs) of *Gn1a* controlling GNPP^13^ and *STRONG CULM2* (*SCM2*) regulating culm thickness and GNPP^14^ genes derived from Habataki were developed in a japonica variety background and evaluated^15^.

The *Grain number 1a* (*Gn1a*) gene was isolated from high-yielding indica rice variety Habataki through QTL analysis with fine mapping analysis^13^. The *Gn1a* gene (*LOC_Os01g10110*) encodes cytokinin oxidase/dehydrogenase2 (OsCKX2), which converts the biological active cytokinin to its inactive form. In cultivar Habataki, the transcription level of *Gn1a* was very low in the developing panicle, probably because of the 16-bp deletion in the 5′-untranslated region (UTR) of the gene, and it caused high cytokinin content in a panicle, resulting in high GNPP. So, the 16-bp deletion was regarded as functional nucleotide polymorphism (FNP), which caused the phenotype. However, the *Gn1a*^Habataki^ allele was also found in high-yielding indica rice cultivar ST12^16^.

The *IPA1/WFP/OsSPL14* gene encoding SQUAMOSA promoter binding protein-like 14 (OsSPL14) was identified through QTL analysis with fine mapping of two independent QTLs by two research groups. *IDEAL PLANT ARCHITECTURE 1* (*IPA1*) was derived from japonica line Shaoniejing^11^ with ideal plant type and high grain number, and *WEALTHY FARMER’S PANICLE* (*WFP*) was derived from high-yielding indica line ST12^16^. *OsSPL14* positively regulates panicle branching and GNPP in the reproductive stage and negatively controls shoot branching (called tillering in rice) in the vegetative stage. Higher expression of the gene in young panicles increased GNPP, resulting in increased yield. Two different yield-positive alleles (*IPA1* and *WFP*) showed strong *OsSPL14* expression in inflorescence organ by two different mechanisms. In line ST12 having the *WFP* allele, *OsSPL14* transcripts were abundant because of less DNA methylation in the *OsSPL14* promoter region compared with that of variety Nipponbare^16^. The *WFP* allele belongs to epigenetic alleles that show a heritable difference in gene expression that is caused by the degree of DNA methylation or chromatin status but not by DNA sequence variations^17^. Recently, another natural epigenetic allele, *ipa1*–*2D*, having tandem repeat sequences of the *OsSPL14* promoter region was also identified^18^. In contrast, the *IPA1* allele (recently renamed as *ipa1–1D* allele by Zhang *et al*.^18^) expressed microRNA (OsmiR156)-resistant *OsSPL14* transcripts because of nucleotide substitution (C to A) at the OsmiR156 target site, resulting in higher expression in panicles^11^. The *IPA1* allele was also found in other japonica varieties (Aikawa1^16^ and Ri22^11^).

Unlike research on genes with qualitative traits, research to test the effect of yield-related genes is more laborious and time consuming. Critical evaluation of the identified genes in different backgrounds in advanced backcross generations will provide solid data about the allele effect and this will be very important information for current rice breeding programs. For this purpose, we launched an evaluation of the effect of yield-related genes of rice in an elite indica cultivar background^19^, which included high-yielding potential breeding lines and popular high-yielding varieties in tropical environments of Southeast Asia and South America. Here, we report the effect of two important genes that regulate grain number, *Gn1a*/*OsCKX2* and *IPA1/WFP/OsSPL14*, in different elite indica cultivar backgrounds in advanced backcross generations. Furthermore, we successfully developed five high-yielding breeding lines using the *OsSPL14*^WFP^ allele through marker-assisted selection (MAS) with conventional breeding strategies.

## Results

### Development of the BC_3_F_2_ populations for the evaluation of *Gn1a*-type 3 allele

**Figure 1:**
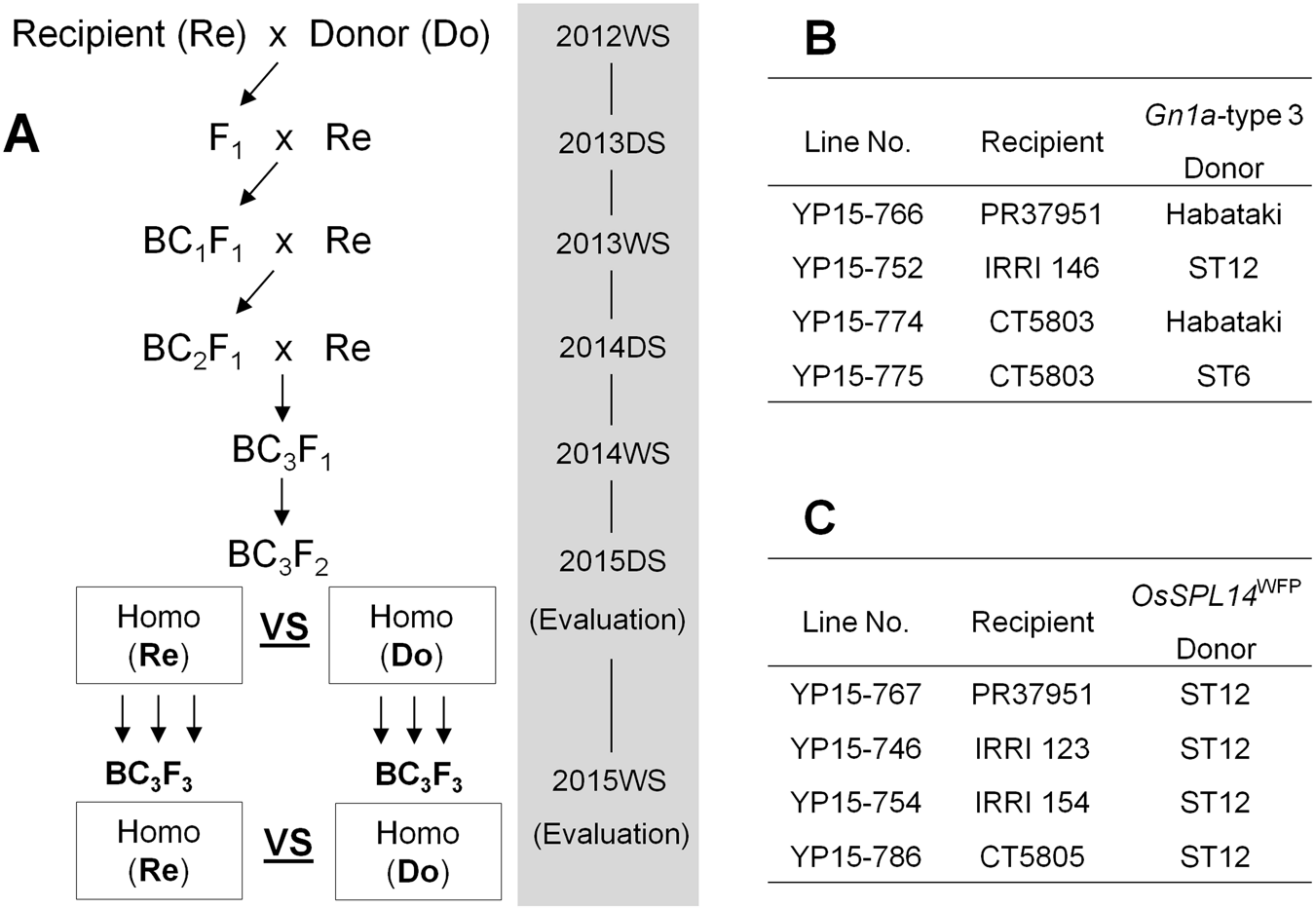
Schematic representation of population development for evaluation of the effect of *Gn1a*-type 3 and *OsSPL14*^WFP^ alleles. (**A**) The recipient (Re) and the donor (Do) were crossed and the true F_1_ plants were selected by SSR markers. Then, the plants having the target allele were selected by marker Gn1a-17 SNP for the *Gn1a*-type 3 allele and by marker SPL14–04 SNP for the *OsSPL14*^WFP^ allele in BC_1_F_1_, BC_2_F_1_, and BC_3_F_1_ generations. A total of 32–50 BC_3_F_2_ plants derived from a single BC_3_F_1_ plant were genotyped by the above markers in each population, and then the mean values of agronomic traits were calculated from each homozygous group in 2015DS. In the following cropping season (2015WS), the phenotype was tested again using 15 BC_3_F_3_ plants derived from three BC_3_F_2_ plants. The year with cropping seasons (WS: wet season, DS: dry season) is depicted in each plant generation. (**B**) Line information for the *Gn1a*-type 3 evaluation. (**C**) Line information for the *OsSPL14*^WFP^ evaluation.

In the beginning of the breeding program, we determined the allele types of yield-related genes from 12 recipient cultivars using whole-genome sequencing and PCR-Sanger sequencing methods, and we further developed allele-specific PCR-gel-based markers for each yield-related gene for MAS^19^. Briefly, the alleles of the *Gn1a* gene were grouped into three types (types 1–3) in the 12 recurrent parents based on the sequences of the *Gn1a* promoter region, which is directly involved in gene expression. Except for tropical japonica cultivar Parao having the *Gn1a*-type 1 allele, all 11 indica cultivars had the *Gn1a*-type 2 allele (IRRI 123, IRRI 146, IRRI 156, IR04A115, PR38012, PR37951, and CT5803) or the *Gn1a*-type 3 allele (IRRI 154, IR05N412, CT5805, and IRGA427). (More information on the recipients appears in the ‘Plant materials’ section below). The crosses between the donor lines and recipients were made for the evaluation of the identified yield-enhancing genes and furthermore breeding of high-yielding lines. So from the same materials, several sub-sets of intermediate breeding lines were randomly selected for the gene evaluation purpose and all materials were advanced to more generations for breeding purpose. The *Gn1a* donor lines, Habataki, ST12, and ST6, had the *Gn1a*-type 3 allele. In this study, we focused on evaluating the effect of the *Gn1a*-type 3 in the recipient cultivar backgrounds having the *Gn1a*-type 2 because the remaining four indica recipient cultivars already had the yield-positive *Gn1a*-type 3 allele. The procedure of the development of near-isogenic lines (NILs) is presented in Fig. 1A. Briefly, the recipient cultivars having the *Gn1a*-type 2 allele were crossed with the *Gn1a* donor lines. The true F_1_ plants were selected by genotyping of polymorphic simple sequence repeat (SSR) markers and they were backcrossed with their recurrent parents, respectively, to remove the donor genome backgrounds. The BC_1_F_1_ plants were genotyped by the allele-specific Gn1a-17 SNP marker and the heterozygous (type 2/type 3) plants were selected for another backcross. With the same procedures, the BC_3_F_1_ plants were produced and self-pollinated to generate homozygous plants for the *Gn1a* locus. For testing of the *Gn1a*-type 3 allele in the diverse recipient genotypes, we selected three different recipients originating from three different institutes (PhilRice, Philippines; IRRI; and CIAT, Colombia). Finally, four BC_3_F_2_ populations (Fig. 1B) were selected and evaluated. To make the same genome status as much as possible except for the *Gn1a* locus, 32–50 BC_3_F_2_ plants derived from a single BC_3_F_1_ plant in each cross combination were genotyped by the Gn1a-17 SNP marker (Supplementary Fig. S1A). The average values of each trait were obtained separately between the *Gn1a*-type 2 and *Gn1a*-type 3 plants.

### Genomic status of the *Gn1a*-type 3 allele-inserted BC_3_F_2_ populations

**Figure 2:**
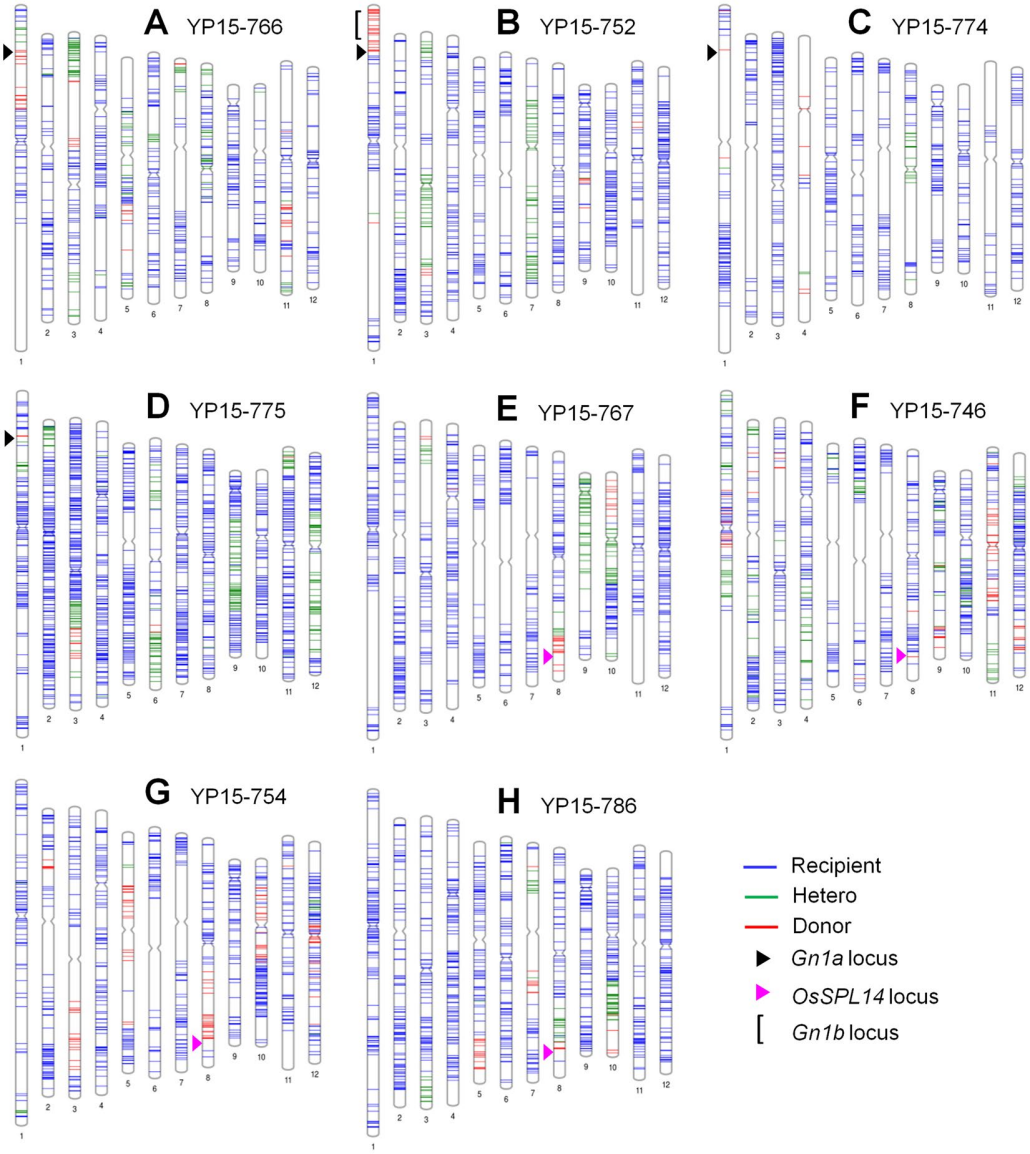
Graphical genotype maps of the BC_3_F_2_ plants. From the eight BC_3_F_2_ populations, one plant per population that had the yield-positive *Gn1a*-type 3 allele (**A–D**) or *OsSPL14*^WFP^ allele (**E–H**) was genotyped by using Infinium 6 K SNP markers. Polymorphic SNPs between bi-parents were used for the construction of the genotype maps.

First, we checked the segregation pattern of the *Gn1a* alleles in BC_3_F_2_ progenies using the chi-square (χ^2^) test. The calculated χ^2^ values were lower than the chi-square critical value 5.991 in *df = *2 and *α = *0.05 conditions, indicating that the *Gn1a* alleles showed a normal Mendelian segregation pattern (recipient allele:heterozygous:donor allele = 1:2:1 ratio) in all backgrounds tested (Supplementary Table S1). One homozygous (type 3/type3) plant from each population was randomly selected and genotyped by high-density SNP markers to check the genome recovery rate of the recurrent parent in the BC_3_F_2_ generation. Of the ~6,000 SNP markers covering 12 chromosomes, 635–1,657 SNPs showed polymorphism between the donor and recipient cultivars, and the selected BC_3_F_2_ plants showed high genome recovery of the recurrent parent by 86.2–97.2% (Supplementary Table S2), which was close to the theoretical expected value of 93.75% for the BC_3_F_1_ generation. Graphical genotype maps were constructed based on the SNP genotyping results (Fig. 2A–D), and it was assumed that the size of the target chromosome segment containing the *Gn1a* locus was 5.5 Mb, 8.6 Mb, and 1.6 Mb in lines YP15–752, YP15–766, and YP15–775, respectively. In the case of line YP15–774, it was difficult to predict the size of the donor segment inherited because of the absence of polymorphic SNPs near the *Gn1a* locus between the parents.

### The *Gn1a*-type 3 allele was not effective on panicle traits in indica cultivars having the *Gn1a*-type 2 allele

**Figure 3:**
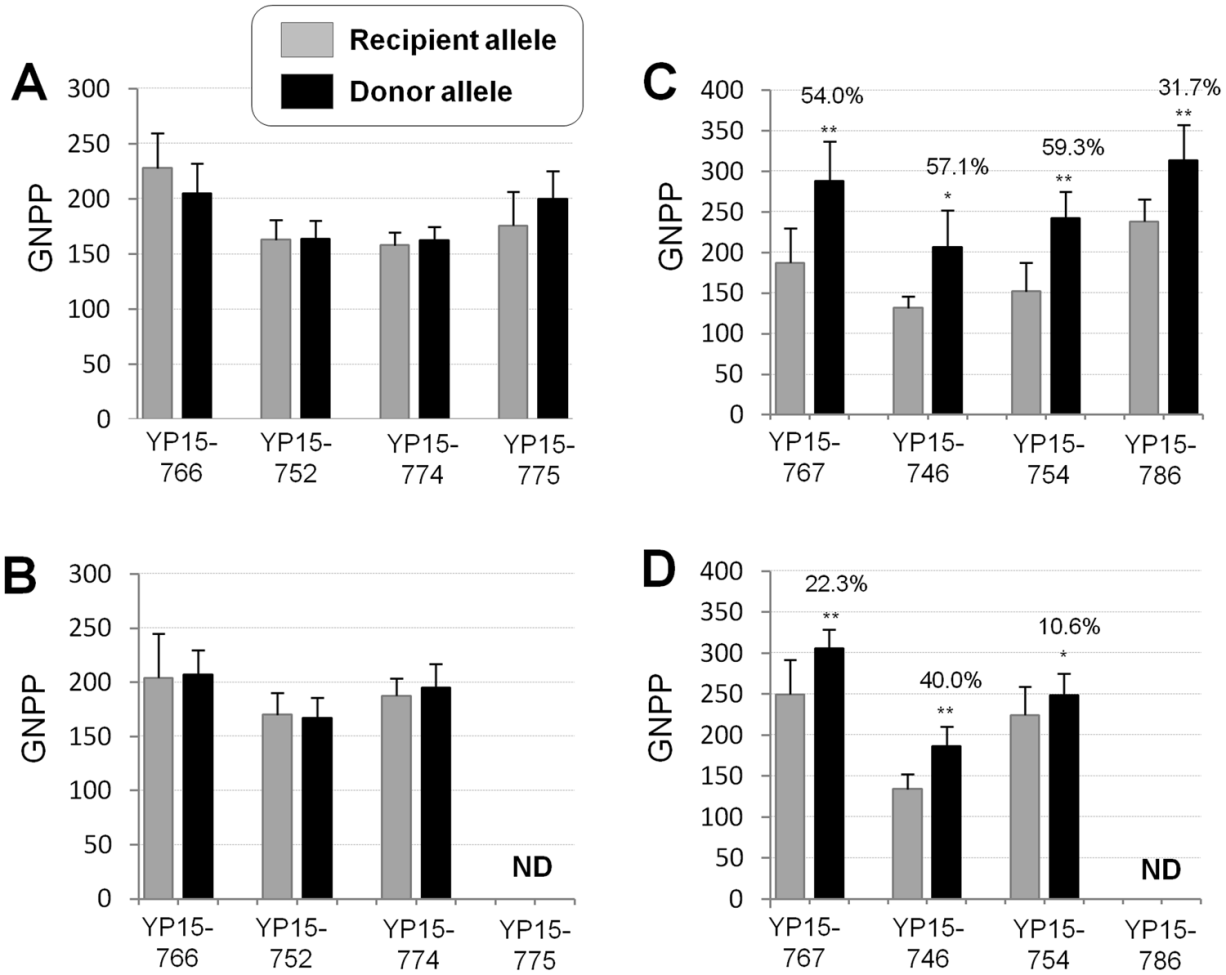
The effect of *Gn1a*-type 3 allele (**A** and **B**) and *OsSPL14*^WFP^ allele (**C** and **D**) in different indica backgrounds. The *Gn1a*-type 3 allele from the different donors (Habataki, ST12, and ST6) was introgressed into three different recipients having the *Gn1a*-type 2 allele. Mean values of GNPP were compared between the *Gn1a*-recipient allele (gray bar) and the *Gn1a*-donor allele (black bar) in the segregating BC_3_F_2_ progenies in each population in 2015DS (**A**) and in BC_3_F_3_ plants in 2015WS (**B**). Similarly, GNPP phenotype was compared between the recipient *OsSPL14* allele and the *OsSPL14*^WFP^ allele in the BC_3_F_2_ generation in 2015DS (**C**) and in the BC_3_F_3_ generation in 2015WS (**D**). The data on lines YP16–775 and YP15–786 were omitted in 2015WS because of rice Tungro virus damage. Asterisks represent a significant difference between two alleles based on Student’s *t*-test (* *α* = 0.05 and ** *α* = 0.01). The effect of the *OsSPL14*^WFP^ allele was shown on the top of the bar as increase rate (%). The error bar means standard deviation.

The mean values of the agronomic traits, including plant height (PH), tiller number per plant (TN), panicle length (PL), primary branching number of panicle (PBN), secondary branching number of panicle (SBN), spikelet fertility (SF), and grain number per panicle (GNPP), were obtained from the *Gn1a*-type 2 and *Gn1a*-type 3 homozygous plants of the BC_3_F_2_ progenies, respectively (*n* = 5–9 plants). Also, the phenotypes of these traits were collected from the recipient cultivars in the same season. Most of the morphological traits were similar to those of their recipient parents (Supplementary Table S3), suggesting that the genome of the tested lines was similar to that of the recipient genome. Our priority traits were PBN, SBN, and GNPP because the *Gn1a* gene was directly associated with these panicle traits. Statistical analysis (*α* = 0.05) showed that there was no significant difference in those panicle trait values between type 2 and type 3 alleles in all four BC_3_F_2_ populations in 2015DS (Fig. 3A; Supplementary Table S3). In the following season (2015WS), the BC_3_F_3_ homozygous plants derived from each BC_3_F_2_ population were tested and the experiments also showed no significant difference in panicle traits (Fig. 3B; Supplementary Table S4). These results suggested that the *Gn1a*-type 3 allele from the different donor sources, including Habataki, ST12, and ST6, was not effective in increasing GNPP in indica varieties PR37951, IRRI 146, and CT5803, which had the *Gn1a*-type 2 allele.

### Development of BC_3_F_2_ populations for evaluating the *OsSPL14*^WFP^ allele and the genome status of the lines

In our breeding program, we used two different donors for yield-positive alleles that strongly express *OsSPL14* in young panicles: indica-type line ST12 harboring less methylated *OsSPL14* promoter (*OsSPL14*^WFP^ allele) and japonica variety Aikawa1 expressing OsmiR156-resistant *OsSPL14* transcript (*OsSPL14*^IPA1^ allele). Both donors were crossed with 12 recipient cultivars and MAS was conducted using marker SPL14–04SNP for detecting the *OsSPL14*^WFP^ allele and marker SPL14–12SNP for the *OsSPL14*^IPA1^ allele^19^. Through the same procedures of *Gn1a* population development, the BC_3_F_2_ populations for the *OsSPL14*^WFP^ allele were developed in four different backgrounds originating from the three different institutes (Fig. 1A and C). Like the *Gn1a* allele assessment, 48–50 BC_3_F_2_ plants derived from a single BC_3_F_1_ plant of each population were genotyped by marker SPL14–04SNP (Supplementary Fig. S1B). The segregation pattern of *OsSPL14* alleles also showed a normal Mendelian pattern (Supplementary Table S1). The background genotyping results showed 909–1,082 polymorphic SNPs between the bi-parents and high genome recovery of the recurrent parent (84.4–93.6%) (Supplementary Table S2). The size of the chromosome 8 segment harboring the *OsSPL14*^WFP^ allele was deduced as 5.5 Mb, 1.6 Mb, 11 Mb, and 2.5 Mb in lines YP15–767, YP15–746, YP15–754, and YP15–786, respectively (Fig. 2E–H).

While we were developing populations using the donor Aikawa1, some unwanted traits such as low fertility and reduced tiller number were observed in most of the recurrent parent backgrounds in the BC_1_F_2_ and BC_2_F_2_ generations (data not shown). Therefore, we could not evaluate the effect of the *OsSPL14*^IPA1^ allele and also no plants having the *OsSPL14*^IPA1^ allele were selected in our breeding program.

### The *OsSPL14*^WFP^ allele greatly improved panicle traits in elite indica backgrounds

The BC_3_F_2_ plants were divided into three groups based on the genotype of the *OsSPL14* alleles and mean values of GNPP were compared between two homozygous groups (the recipient and ST12 alleles). GNPP was much higher (31.7–59.3%) in NILs having the *OsSPL14*^WFP^ allele than with the recipient allele in all four BC_3_F_2_ populations in 2015DS (Fig. 3C). Without a change in PL, both PBN and SBN increased significantly with the *OsSPL14*^WFP^ allele in the four indica cultivars, resulting in increased GNPP (Supplementary Table S3). Phenotypic inheritance of panicle traits was also observed in the following generation BC_3_F_3_ in 2015WS (Fig. 3D; Supplementary Table S4). These results suggested that the genetic effect of the *OsSPL14*^WFP^ allele was very strong in diverse indica backgrounds and stable across cropping seasons.

### Development and evaluation of pyramiding lines having both *Gn1a*-type 3 and *OsSPL14*^WFP^ alleles

The *Gn1a*-type 3 and *OsSPL14*^WFP^ alleles were pyramided in two indica backgrounds, IRRI 123 and IRRI 156, respectively, to test the additive effect of *Gn1a* and *OsSPL14* genes. The procedures of line development are shown in Supplementary Fig. S2. Through repetitive backcrosses with MAS, we finally selected a single heterozygous plant having both genes in an advanced generation, and the plant was self-pollinated to generate homozygous progenies. Approximately 200 plants per population were genotyped by both Gn1a-17 SNP and SPL14–04 SNP markers. Allele segregation of the two genes was tested using the chi-square method and the calculated χ^2^ values were lower than the chi-square critical value of 15.507 in *df = *8 and *α = *0.05 conditions, indicating that both genes showed a normal Mendelian segregation of two independent loci (Supplementary Table S5). Agronomic traits were evaluated and the mean values were calculated among the same genotypic plants in 2016DS. As we observed previously in single-gene evaluation, there was no significant effect of the *Gn1a*-type 3 allele on panicle traits in both backgrounds (Table 1). In contrast, the *OsSPL14*^WFP^ allele dramatically elevated GNPP by 29.8% and 60.8% in lines YP16–18 and YP16–02, respectively. The two yield-gene-combined groups (*Gn1a*-type 3/*OsSPL14*^WFP^) showed the highest GNPP, but it was not significantly different from the single-gene *OsSPL14*^WFP^ groups in both backgrounds (*α* = 0.05) (Table 1). This result suggested that the effect of the *Gn1a*-type 3 allele was absent in the *Gn1a*-type 2 background and this led to no significant additive effect in the two-gene combined lines.

**Table 1:** Agronomic traits of the combined lines with two yield-enhancing genes.

Line	Alleles		PH (cm)	TN	PL (cm)	PBN	SBN	GNPP	TGW (g)
	*Gn1a*	*OsSPL14*							
YP16–02	Recipient	Recipient	93.3^a^	10.0	24.7	11.9^a^	39.3^a^	147.0^a^	18.1
	Type 3	Recipient	104.2^b^	11.6	26.9	12.6^a^	46.8^a^	167.9^a^	22.1
	Recipient	*OsSPL14* ^WFP^	90.8^a^	9.8	25.0	16.0^b^	58.9^b^	236.4^b^	19.0
	Type 3	*OsSPL14* ^WFP^	106.6^b^	10.6	27.2	17.1^b^	70.7^c^	262.3^b^	21.6
YP16–18	Recipient	Recipient	93.6	12.2^a^	25.0	11.1^a^	45.3^a^	165.3^a^	20.3
	Type 3	Recipient	90.8	12.6^a^	25.9	11.2^a^	49.0^a^	179.0^a^	21.5
	Recipient	*OsSPL14* ^WFP^	96.8	7.4^b^	22.4	14.2^b^	60.0^b^	214.5^b^	20.1
	Type 3	*OsSPL14* ^WFP^	96.2	9.2^b^	23.2	14.6^b^	65.9^b^	237.2^b^	19.8

Significant difference was depicted by the different letters (a, b, and c) followed by values (Duncan’s multiple-range test, *α* = 0.05; *n* = 5 plants). The background variety of the lines YP16–02 and YP16–18 was IRRI 123 and IRRI 156, respectively. PH: plant height, TN: tiller number, PL: panicle length, PBN: primary branching number of panicle, SBN: secondary branching number of panicle, GNPP: grain number per panicle, TGW: 1,000-grain weight.

### Breeding of high-yielding lines using the *OsSPL14*^WFP^ allele

**Figure 4:**
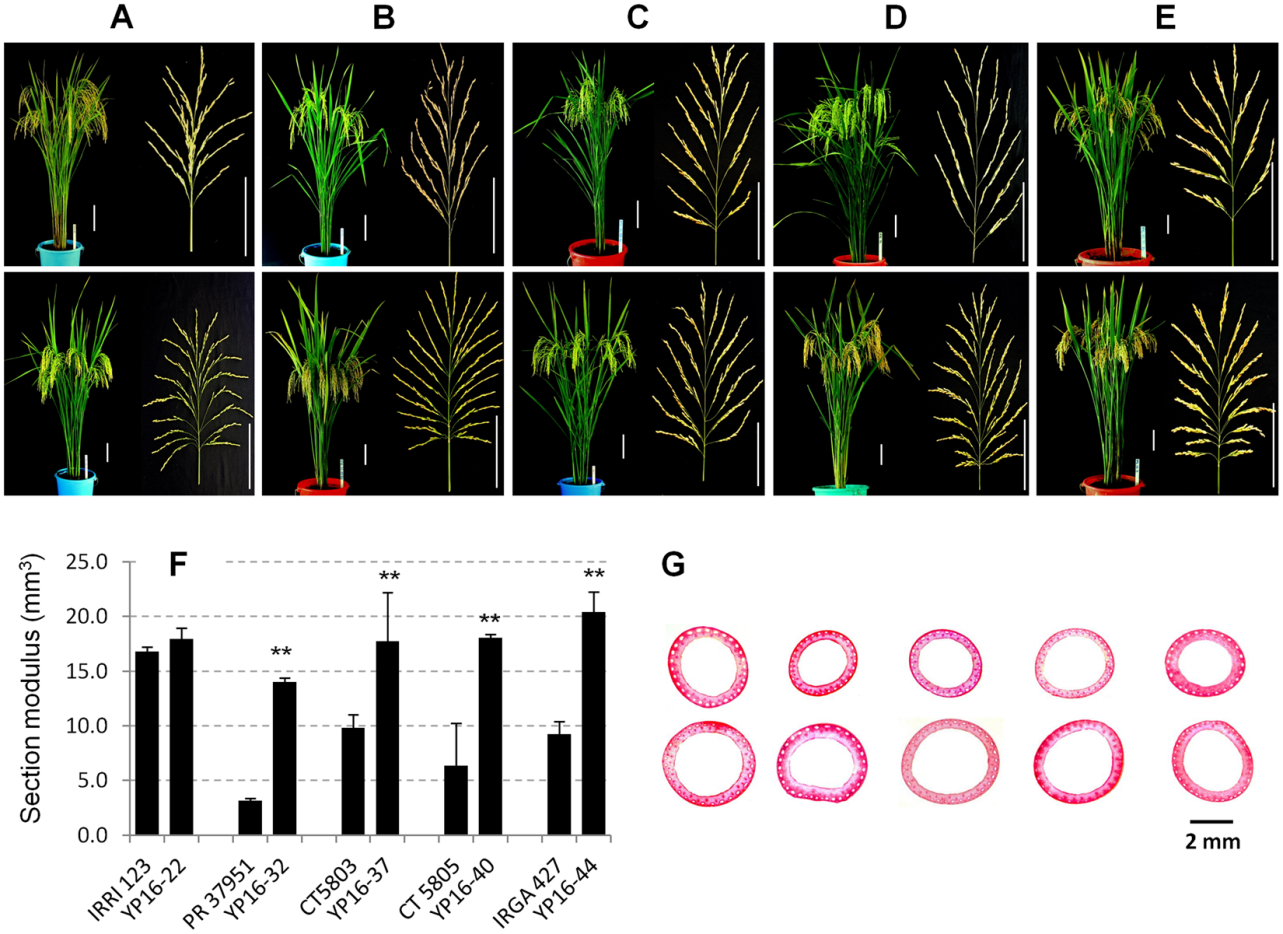
Morphological analyses of the selected five high-yielding lines with their recipients. (**A–E**) Plant and panicle images from the representative plant of the recurrent line (top) and the improved line (bottom). Scale bar = 10 cm. (**A**) IRRI 123 (top) and YP16–22 (bottom). (**B**) PR37951 and YP16–32. (**C**) CT5803 and YP16–37. (**D**) CT5805 and YP16–40. (**E**) IRGA427 and YP16–44. (**F**) Section modulus (SM) values of the third internode. Student’s *t*-test (* *α* = 0.05 and ** *α* = 0.01) was used. The error bar means standard deviation. (**G**) Cross-section images of the fourth internodes. Sample order is consistent with the above plant/panicle images.

To develop high-yielding breeding lines, we used combined breeding approaches of MAS and phenotypic selection because, as a general phenomenon in MAS breeding, the target-gene-inserted lines frequently exhibit unwanted agronomic traits, which might be caused by undesirable genome reconstitution of the parents. Our combined breeding scheme with pedigree is described based on the selected five high-yielding lines (Supplementary Fig. S3). Briefly, after crossing between the donor and recipient lines, MAS was conducted for the target genes in each backcross generation. In advanced generations, plant selection was conducted from the grain filling stage to maturity stage in the field, and finally 57 promising plants were selected from more than 680 breeding lines in 12 recipient backgrounds based on MAS and our phenotype selection criteria, including high spikelet fertility, high grain filling rate (heavy panicles), less variation among tillers, slow plant senescence process, and high lodging resistance, in 2015DS. Further, the selected plants were self-pollinated to fix the genomes in 2015DS/WS, and finally a preliminary yield trial was conducted with 20 promising breeding lines in 2016DS. Among them, the best five breeding lines (YP16–22, YP16–32, YP16–37, YP16–40, and YP16–44) in the backgrounds of IRRI 123, PR37951, CT5803, CT5805, and IRGA427, respectively, were selected (Supplementary Table S6) and further fixed in 2016WS. Background genotyping showed high genome recovery of the recipient genome with the *OsSPL14*^WFP^ allele in all five selected lines. In lines YP16–22 and YP16–32, the *Gn1a*-type 3 allele was also introgressed from the donor lines (Supplementary Fig. S4). From the selected five breeding lines, grain yield and major agronomic traits were measured and compared between the recipient and improved lines as well as between the high-yielding check varieties and the improved lines in 2017DS. The overall plant phenotypes of the breeding lines were similar to those of each background recipient except for a few traits (Fig. 4A–E, Table 2). Like some different phenotypes, line YP16–22 flowered ~12 days earlier than its recurrent parent variety, IRRI 123, but the other breeding lines exhibited the same heading date as their recurrent parents. Interestingly, PH increased significantly by 7–17 cm except for line YP16–22. A minor tendency of TN reduction was observed in the breeding lines. In addition, the harvest index (HI) value was higher in the breeding lines except for line YP16–40. However, the most drastic phenotypic change was for panicle traits. PBN, SBN, and GNPP increased significantly (*α* = 0.01) by 29.4–85.4%, 63.3–165.2%, and 56.6–202.1%, respectively, compared to their background materials (Table 2). This might be caused by the effect of the *OsSPL14*^WFP^ allele as we observed in our previous experiments. In addition, the grain yield of the plot (10 m^2^) increased markedly by 28.4–83.5% in comparison with that of the improved line and its background. The main reason for the yield enhancement was probably a major trait change in the breeding lines, which was GNPP. Grain yield was also compared with that of the current top-yielding tropical varieties (IRRI 146, IRRI 154, and IRRI 156). They produced 5.79–7.82 t/ha in our experiment. Except for line YP16–22, all four breeding lines showed higher yield (23.4–64.7%) than IRRI 156, which was the highest yielder among the checks in our trials (Table 2).

**Table 2:** Yield and agronomic traits of the selected high-yielding lines.

Plant material^a^	DTH	PH (cm)	TN	PL (cm)	PBN	SBN	GNPP	SF (%)	TGW (g)	HI	Grain yield^b^ (t/ha)	Yield (%) to RP^c^	Yield (%) to IRRI 156^d^
IRRI 123	88.5	89.0	14.3	23.7	9.7	31.0	117.8	88.7	23.9	0.476	6.12 ± 0.57		78.3
YP16–22	76.0^*^	87.1	8.7^**^	24.5	17.4^**^	82.2^**^	340.9^**^	83.5^*^	22.8	0.570^**^	7.87 ± 0.79	128.4	100.5
PR37951	93.0	100.3	11.1	27.2	9.5	35.9	135.1	77.9	24.5	0.474	6.89 ± 0.29		88.1
YP16–32	91.0	107.3^**^	10.3	27.0	14.7^**^	94.2^**^	408.0^**^	85.8^*^	22.1^**^	0.520	9.66 ± 1.72	140.1	123.4
CT5803	89.0	91.0	12.9	26.6	10.0	35.0	151.0	93.2	26.2	0.458	6.95 ± 0.38		88.8
YP16–37	87.5	108.8^**^	11.7	29.4^**^	12.9^**^	62.3^**^	236.4^**^	89.6^**^	26.6	0.549	11.53 ± 0.52	166.1	147.4
CT5805	99.5	98.3	10.4	28.1	11.7	60.8	218.3	88.6	24.1	0.503	7.02 ± 0.47		89.7
YP16–40	96.5	107.0^**^	9.8^*^	29.0^*^	19.3^**^	99.3^**^	379.6^**^	88.2	23.3	0.469	12.89 ± 0.25	183.5	164.7
IRGA427	96.5	83.6	12.2	20.7	8.9	30.4	118.6	91.1	23.9	0.488	6.72 ± 0.85		85.9
YP16–44	97.5	105.0^**^	11.5	24.6^**^	16.5^**^	66.3^**^	263.6^**^	91.0	24.1	0.512	10.79 ± 0.45	160.7	138.0
IRRI 146	95.0	79.7	13.4	22.0	10.0	31.6	139.0	85.0	20.6	0.510	5.79 ± 0.39		74.0
IRRI 154	95.0	93.4	12.7	25.9	10.2	44.4	155.9	90.4	23.0	0.511	7.00 ± 0.80		89.4
IRRI 156	97.0	93.5	13.5	25.1	9.9	30.7	110.9	84.6	23.9	0.485	7.82 ± 0.58		100.0

^a^Five high-yielding lines (YP16–22, YP16–32, YP16–37, YP16–40, and YP16–44) were compared with their background cultivars (IRRI 123, PR37921, CT5803, CT5805, and IRGA427), respectively. Additionally, three high-yielding varieties in the Philippines were included as check varieties.

^b^Grain yield of a 10-m^2^ plot was converted to tons per hectare (t/ha). Mean values with standard deviation were obtained from two replications in 2017DS.

^c^Percentage yield compared to each recurrent parent (RP).

^d^Percentage yield compared to the highest check variety, IRRI 156.

Significant difference between the breeding line and its recurrent parent was calculated based on Student’s *t*-test (* *α* = 0.05 and ** *α* = 0.01). DTH: days to heading, PH: plant height, TN: tiller number, PL: panicle length, PBN: primary branching number of panicle, SBN: secondary branching number of panicle, GNPP: grain number per panicle, SF: spikelet fertility, TGW: 1,000-grain weight, HI: harvest index.

Lodging resistance is one of the important agronomic traits, especially in high-yielding lines to physically support their high grain yield. We measured the physical parameters of the main culm and these values were converted to the value of section modulus (SM), which was formulated by Ookawa *et al*.^14^. The SM value of the third internode was significantly higher than that of the recipients (Fig. 4F). Also, culm diameter of the fourth internode was higher in all five breeding lines (Fig. 4G). In addition, there was no significant lodging damage based on our observations in the field for three cropping seasons (2016DS, 2016WS, and 2017DS). This physical culm strength and our observations suggest that the breeding lines have some degree of lodging resistance.

Among all the yield trial materials, the highest grain yield was obtained from line YP16–40 (BC_2_F_7_ generation), which was derived from the cross CT5805 x ST12 (Supplementary Fig. S3D). This breeding line showed significant improvement in yield by 83.5% compared with that of recipient cultivar CT5805 and by 64.7% compared with that of the highest yielding check variety, IRRI 156 (Table 2). This line had remarkable improvement in plant architecture such as dark green V-shaped erect leaves, long flag leaf, and thick culm (Supplementary Fig. S5, Fig. 4F,G). The plants stood up straight even though the panicles became heavy at the full maturity stage in three cropping seasons. These traits might also be involved in grain yield increase. The background genotyping analysis detected introgression of a few donor DNA segments from chromosomes 2, 3, 5, and 8, including the *OsSPL14* locus (Supplementary Fig. S4D). Interaction between these donor DNA segments and the recipient genome improved plant architecture and hence grain yield.

## Discussion

Increased rice production per unit area is the mandate to feed the increasing population and to achieve global food security in the near future. However, the genetic gain in rice yield potential has stagnated for several decades, especially in the tropical rice-growing regions of Asia^20,21^. A recent report by Dingkuhn *et al*.^21^ reflects the stagnation in yield potential, in which simultaneous yield evaluation of 12 elite irrigated rice cultivars, including old and current varieties in the Philippines, showed that the current best tropical rice varieties do not have higher yield potential than some old popular varieties developed in the 1970s and 1980s. In addition, although many yield QTLs/genes are cloned in rice, those genes have not been reported to increase the grain yield of indica rice cultivars in the field^6^. Recently, one gene, *SPIKLET NUMBER* (*SPIKE*), originating from a tropical japonica landrace has shown a high possibility to increase yield potential in indica rice backgrounds^22^. The identification of new yield-enhancing genes, critical evaluation of cloned genes, and the application of effective alleles in actual breeding programs are expected to improve the genetic yield potential of elite rice cultivars. In this study, we presented a method for allele evaluation and allele application for breeding. Interactions between the QTLs/genes and genetic backgrounds and between the QTLs/genes and environment have been reported in many vegetables and cereals^23–26^. For the clear evaluation of the effect of yield-enhancing alleles, the same yield-positive alleles of *Gn1a* and *OsSPL14* genes were tested in different indica cultivar backgrounds and in two cropping seasons. Moreover, to exclude the influence of other donor DNA segments, the alleles’ effects were compared at similar genome status except for the target locus by using segregating BC_3_F_2_ progenies, which were derived from a single heterozygous BC_3_F_1_ plant. Also, background genotyping by high-density SNP markers provided clearer information on the genome reconstitution of parents in the breeding lines than the conventional low-density SSR markers. Our evaluation method may produce solid data on allele mining effects and will be applicable for evaluating other trait-related genes.

In previous reports, the *Gn1a*^Habataki^ (type 3) allele significantly increased GNPP by 21% and 28~37% in temperate japonica cultivar (Koshihikari and Sasanishiki) backgrounds, respectively^13,15^. In addition, the *Gn1a*-indica allele increased grain yield in Kongyu 131^27^ with elite japonica background. However, in our study, the *Gn1a*-type 3 allele derived from lines Habataki, ST12, and ST6 was not effective in several indica cultivar backgrounds that had the *Gn1a*-type 2 allele. These results indicate that the *Gn1a*-type 2 allele has the same functionality with the *Gn1a*-type 3 allele although three SNPs were found in the promoter region between the two alleles^19^. As reported in a previous study^13^, the 16-bp deletion found in both alleles might be directly associated with GNPP rather than the three SNPs. However, the expression level of *Gn1a* in young panicles needs to be compared among the three allele types in a further study. The allele of the *Gn1a* gene with high GNPP was originally identified from indica-type variety Habataki^13^. Habataki is a Japanese high-yielding variety derived from a cross between two Korean Tongil-type breeding lines, Milyang 42 and Milyang 25^28^. More than 90% of the Tongil genome is derived from indica parents^29^. The pedigree of Habataki^28^ suggests that variety Habataki might have much indica genomes, probably IR8 (Green Revolution variety) and IR24 developed by IRRI, which were widely distributed in East Asian countries, and those were also used as breeding sources of the modern indica rice cultivars^30^. This information suggests that the *Gn1a*^Habataki^ allele might originate from high-yielding indica lines and the allele might be continuously selected in indica rice breeding programs. This might be the cause of a yield plateau in modern indica rice cultivars. Consistent with this, out of our 12 recipient cultivars, four had exactly the same allele as Habataki at the sequence level^19^ and another seven indica varieties had the *Gn1a*-type 2 allele, while the other one had the *Gn1a*-type 1 allele. The yield-positive *Gn1a* allele was also found in Chinese high-yielding indica varieties 93–11 and Teqing^26^. Hence, most of the elite indica varieties might already have yield-positive *Gn1a* alleles (type 2 or type 3), and these indica alleles will be effective in many japonica cultivars having the *Gn1a*-type 1 allele, as shown in previous reports. *Gn1a* together with *Gn1b* (another QTL near the *Gn1a* locus in Habataki) exhibited an additive effect in GNPP by ~45% in a Koshihikari background^13^. In line YP15–752, we found that the edge of the short arm of chromosome 1 (~5.5 Mb segment) containing both *Gn1a* and *Gn1b* loci was derived from Habataki (Fig. 2B). But, GNPP did not increase significantly in this line, suggesting that the *Gn1b* allele might also be the same between Habataki and the background of YP15–752 of IRRI 146.

In this study, two different yield-positive alleles of the *OsSPL14* gene were tested in indica backgrounds. One of the donors, japonica variety Aikawa1, showed high GNPP with very low TN (~3 tillers per plant) in an IRRI field in the Philippines. Some of our breeding lines derived from Aikawa1 also exhibited very low TN with some other undesirable agronomic traits, which might be caused by an indica x japonica cross. Therefore, for the critical evaluation of the *OsSPL14*^Aikawa1^ allele, removing the donor genome in the breeding lines is needed for further study. However, a severe reduction in TN (45.3%) was also reported in the NIL *OsSPL14*^IPA1^ in the japonica background of Xiushui 11^11^. Therefore, breeders need to consider the effect of the *OsSPL14*^IPA1^ allele on the trait tiller number. Unlike Aikawa1, cultivar ST12 produced approximately 12 tillers per plant in the IRRI field. Also, there was no severe reduction in TN in our NILs having the *OsSPL14*^WFP^ allele. Thus, the NILs and the newly bred high-yielding lines produced 8.7~11.7 tillers per plant in different backgrounds. However, a minor tendency of a reduction in TN was observed in some backgrounds (Tables 1 and 2; Supplementary Tables S3, S4, and S6). But, this reduction is not of a considerable amount in current breeding programs, and we successfully improved grain yield from the five elite indica backgrounds using the *OsSPL14*^WFP^ allele. The *OsSPL14*^WFP^ allele significantly increased PBN and SBN, which resulted in high GNPP in all high-yielding lines with an indica background tested across cropping seasons. Similarly, this allele also improved GNPP (51.3%) in Nipponbare with a japonica background^16^. These data suggest that the *OsSPL14*^WFP^ allele can provide a strong genetic gain in grain production across diverse genetic backgrounds and environments. Recent molecular studies revealed the function of IPA1/WFP/OsSPL14 in both shoot and panicle branching. IPA1/WFP/OsSPL14 transcription factor binds directly to the promoter sequences of the key regulators of tillering, *OsTB1*, and of panicle architecture, *DEP1*^31^. In addition, IPA1/WFP/OsSPL14 protein level is regulated by IPA1 INTERACTING PROTEIN1 (IPI1) in a tissue-specific ubiquitination manner and the *ipi1* mutant showed both increased tiller number and panicle size^32^. IPA1/WFP/OsSPL14 together with DWARF 53 protein is the key repressor of the strigolactones signaling pathway which controls tillering in rice^33^. These reports suggest that IPA1/WFP/OsSPL14 plays a key role in two major architecture traits, tillering and panicle branching. The newly identified natural epigenetic allele *OsSPL14*^ipa1–2D^ having tandem repeat sequences of *OsSPL14* promoter region exhibited high GNPP without a tiller reduction^18^. Therefore, the application of proper alleles of *OsSPL14* can improve plant architecture. Furthermore, the identification of new functional alleles of the known yield-related genes may extend breeders’ choice in attempting to increase yield.

To enhance trait performance using an additive effect of different genes, gene pyramiding in the same background is widely used in MAS breeding programs. In japonica variety Nipponbare possessing the *Gn1a*-type1 allele, lines combined with the two genes (*Gn1a*-type 3 and *OsSPL14*^WFP^) showed higher GNPP (~70.4%) than single-gene introgressed lines with *Gn1a* by 21.5% and with *OsSPL14*^WFP^ by 51.3%, respectively^16^. However, we could not see a significant additive effect of two genes in indica backgrounds because of the absence of a *Gn1a*-type 3 effect in *Gn1a*-type 2 backgrounds (Table 1).

Our combined breeding approach was successful in breeding high-yielding lines. Five selected lines showed higher grain yield than their recipient cultivars as well as current top-yielding tropical rice cultivars such as IRRI 146, IRRI 154, and IRRI 156, which were also included as high-yielding checks in the experiment on yield comparison between old and current tropical varieties by Dingkuhn *et al*.^21^. Yield improvement of our breeding lines might be possible because of the strong effect of the *OsSPL14*^WFP^ allele and other improved agronomic traits driven by phenotypic selection. One remarkable common phenotype among five breeding lines is increased stem diameter (Fig. 4F–G). Similarly, the *OsSPL14*^ipa1–2D^ allele also promoted culm diameter^18^, suggesting that the *OsSPL14*^WFP^ allele might increase stem thickness. Four breeding lines showed increased PH. This trait, providing a little bit larger plant size, might also influence yield increase. Another character, HI, might be involved in yield enhancement. A positive correlation between grain yield and HI was reported in rice^34,35^. Overall, our breeding lines showed a higher HI value than the recurrent parents (Table 2). Yield improvement was more remarkable in varieties with high yield potential of Latin America^36^, including CT5803, CT5805, and IRGA427, suggesting that the genetic combining ability of ST12 was much better with Latin American rice genotypes than IRRI high-yielding indica cultivars. These high-yielding lines need to be tested in Latin American countries in the future. In our breeding program, we could not obtain very good breeding lines in other IRRI variety backgrounds such as IRRI 146, IRRI 154, and IRRI 156. In these backgrounds, GNPP increased but actual yield did not improve significantly because of other negative agronomic traits. Currently, precise molecular breeding is available because of low-cost genomics approaches. To remove undesirable phenotypes, we need to further eliminate the donor DNA segments and/or trim the surrounding sequences of the *OsSPL14*^WFP^ gene. In this study, the size of the introgressed DNA segment containing the target locus was higher than 1.6 Mb (1.6–11.0 Mb) (Fig. 2). This can lead to linkage drag in some specific backgrounds. However, for precise MAS, recombinant selection in early breeding generations is required. High-density background genotyping using genome-wide SNP markers or next-generation sequencing techniques provide a precise genome map of the breeding lines and enable us to know the number and size of the chromosome segments derived from the donor line. Eventually, the donor genome rate will be decreased by genotyping of the anchored donor-removing/donor-trimming with additional backcrossing and selfing. Through this process, we can expect actual yield improvement from IRRI top-yielding variety backgrounds.

In terms of rice plant architecture, long, erect, and V-shaped leaves will be beneficial to capture light energy, resulting in high photosynthetic efficiency and high grain yield^37–39^. Line YP16–40 generated erect V-shaped leaves, elongated flag leaf, and dark green leaves (Supplementary Fig. S5), which were probably caused by parental genome effects or epistasis between the parental genomes. This ideal leaf morphology needs to be tested in a further study to enhance yield.

To improve genetic yield potential, the identification of new yield-enhancing genes is important. Along with this, critical evaluation of the identified genes in several elite backgrounds and pyramiding of effective alleles in the same background are also crucial. Here, we showed a strong possibility to increase rice grain yield in elite indica backgrounds using the *OsSPL14*^WFP^ allele. The *IPA1/WFP/OsSPL14* gene may emerge as a new Green Revolution gene after the semi-dwarf (*sd1*) gene because of its importance in expressing improved rice plant architecture^40^. The epigenetic *OsSPL14*^WFP^ allele is unique throughout rice germplasm and so this allele will be effective in most rice cultivars, including both indica and japonica types. We believe that this allele upgrades the current stagnant yield potential of tropical indica varieties and also contributes genetic yield enhancement in many favorite local varieties from different countries.

## Methods

### Plant materials

This new frontier breeding project began in 2012 under the Global Rice Science Partnership (GRiSP) program to increase the genetic yield potential of 12 elite indica cultivars using molecular breeding of eight identified yield-related genes: *Gn1a/OsCKX2*, *IPA1/WFP/OsSPL14*, *SCM2*, *SPIKE*, *DEP1*, *qSW5*, *GS5*, and *TGW6*. The donor lines of yield-related genes and recipient lines were listed in our previous report^19^. In this study, three cultivars (Habataki, ST12, and ST6) were used as donors for the *Gn1a*-type 3 allele and ST12 and Aikawa1 were used as donors for the *OsSPL14*^WFP^ and *OsSPL14*^IPA1^ allele, respectively. As recipients, 12 high-yielding breeding lines or high-yielding popular varieties were used. Two high-yielding indica lines, PR37951–3B-37–1–2 (presented as PR37951) and PR38012–3B-3–1 (presented as PR38012) from the Philippine Rice Research Institute (PhilRice) of the government of Philippines; two high-yielding indica lines (IR04A115 and IR05N412) from the IRRI breeding program; four elite high-yielding popular cultivars, IRRI 123 (PSB Rc82), IRRI 146 (NSIC Rc158), IRRI 154 (NSIC Rc222), and IRRI 156 (NSIC Rc238) developed by IRRI; and four cultivars with high yield potential from Latin America^36^, CT5803 (CT19021–3–4–1V1–1), CT5805 (CT21375-F4–43–1), IRGA427, and Parao, obtained from the International Center for Tropical Agriculture (CIAT), Colombia, were used in this study. The final five high-yielding breeding lines (YP16–22, YP16–32, YP16–37, YP16–40, and YP16–44) were assigned with the IRRI designation numbers IR15W1002, IR15W1003, IR15W1004, IR15W1005, and IR15W1006, respectively. All crosses and line development were conducted at IRRI, Los Baños, Philippines (14°11′N, 121°15′E) from the 2012WS to 2017DS. In every season, the breeding lines were grown with their recipient materials in the same experimental field at IRRI. All the experimental protocols and methods used in this study complied with the institute guide lines and regulations.

### Phenotype measurement of agronomic traits

Various agronomic traits, including DTH (days to heading), PH, TN, PL, PBN, SBN, GNPP, SF, and TGW (1,000-grain weight), were manually measured. DTH was counted from seed sowing to the actual 50% flowering of the plants of each line. The reproductive tillers having panicles with filled grains were counted for TN. TGW was determined by measuring the weight of harvested seeds that were air-dried in a glasshouse and oven-dried at 50 °C until they reached ~14.0% moisture content. Panicle-related traits containing PL, PBN, SBN, GNPP, and SF were measured from five tillers and three tillers per plant in BC_3_F_2_ and BC_3_F_3_ generations, respectively.

### Yield trials and harvest index (HI)

The selected advanced breeding lines of BC_1_F_7_, BC_2_F_7_, and BC_1_F_8_ generations with their respective recurrent parents and high-yielding check varieties were evaluated for grain yield and yield-related traits in 2017DS to compare their yield performance in a randomized complete block design in two replications. One seedling per hill was manually transplanted at 20 cm × 20 cm spacing between hills and rows. Two rows of purple rice plants were planted surrounding the test materials. Fertilizer management was as follows: nitrogen (urea) at the rate of 60–60–60–20 kg/ha (basal-21 DAT-42 DAT-flowering), phosphorus at 30 kg/ha using solophos, potassium (muriate of potash) at 40 kg/ha, and zinc (zinc sulfate) at 5 kg/ha. Plot yield represents grain yield (14% moisture content) of a 10-m^2^ plot (250 plants). Other agronomic traits (DTH, PH, TN, PL, PBN, SBN, GNPP, SF, and TGW) were measured from the high-yielding lines and recipients. Harvest index (HI) was determined based on the method described by Laza *et al*.^35^. Briefly, shoots and grains from the plants harvested in a 0.48-m^2^ area (12 hills) were separated and dried in an oven at 70 °C for 4–5 days. Then, HI was calculated by using the following formula: HI = (grain yield)/(grain yield + straw dry weight).

### Measurement of stem phenotype

The third and fourth internodes of the main culm were harvested from five plants at maturation stage (20 days after heading) and the samples were fixed in 1:1:18 FAA (35% formalin:glacial acetic acid:70% alcohol) solution. Cross-sectioned culm tissues were stained with safranin red and observed under a Zeiss Axioplan stereomicroscope. The physical parameter of the culm, section modulus (SM), was calculated by using the formula of Ookawa *et al*.^14^: SM = π/32 × (*a*_1_^3^*b*_1_ − *a*_2_^3^*b*_2_)/*a*_1_, where *a*_1_ is the outer diameter of the minor axis in an oval cross-section, *b*_1_ is the outer diameter of the major axis in an oval cross-section, *a*_2_ is the inner diameter of the minor axis in an oval cross-section, and *b*_2_ is the inner diameter of the major axis in an oval cross-section.

### DNA preparation and PCR genotyping for MAS

Leaf samples were collected from the individual plants of intermediate breeding lines and parents. Genomic DNA was prepared by using the simple DNA preparation method^41^, which does not require phenol/chloroform extraction and isopropanol precipitation steps. Briefly, a small piece (about 4 cm long) of fresh leaf or stored leaf at −20 °C was ground using a 2010 Geno/Grinder (www.spexsampleprep.com) with the help of liquid nitrogen. In each tube, 200 µL of TPE buffer (100 mM Tris-HCl pH 9.5, 1 M KCl, 10 mM EDTA pH 8.0) were added and the samples were incubated at 65 °C for 30 min. The samples were diluted by adding 1 mL of water and centrifuged for 10 min at the maximum speed. The supernatant was directly used as template DNA for PCR genotyping. For MAS, we used the previously developed allele-specific markers of Kim *et al*.^19^: marker Gn1a-17 SNP for *Gn1a*-type 3 allele, marker SPL14–04 SNP for the *OsSPL14*^WFP^ allele, and marker SPL14–12 SNP for the *OsSPL14*^IPA1^ allele. The 20-µL PCR solution contained 1× PCR buffer, 200 μM of each dNTP, 0.25 μM of each primer, 1.5 µL of leaf extract prepared by the TPE method, and 1 unit of *Taq* DNA polymerase. Thermal cycles were programmed as follows: 94 °C, 3 min; 35 cycles of 95 °C for 25 s; 55 °C for 25 s; and 72 °C for 35 s, concluding with 72 °C for 5 min. The PCR products were analyzed in 2.5% agarose gel.

### Background genotyping and construction of a graphical genotype map

Genomic DNA was prepared by the modified CTAB method^42^. Background genotyping was conducted in the IRRI Genotyping Service Laboratory (http://gsl.irri.org/) by using high-density SNP markers, which were placed on the Illumina Infinium 6 K SNP chip. The SNP genotyping was preceded by DNA hybridization to the probes on the chip, single nucleotide (target SNP) extension, and fluorescence detection steps based on the manufacturer’s instructions. The SNP genotype data were analyzed in Microsoft Excel software to remove the failed SNP calling and monomorphic SNP data points between parents. The graphical genotype map was constructed based on the selected polymorphic SNP data using the web tool PhenoGram^43^ (http://visualization.ritchielab.psu.edu/). All methodologies used for molecular genotyping and PCR analysis were approved by the committee of the Institute and the government of the Philippines. The experiments were conducted in accordance with the relevant guide lines and regulations.

### Statistical analysis

All phenotypic data were recorded on a Microsoft Excel sheet. Phenotypic comparisons between the target allele and non-target allele for *Gn1a* and *OsSPL14* genes were performed using Student’s *t*-test in Microsoft Excel software. Multiple comparisons were analyzed by Duncan’s multiple range test (DMRT) using the Statistical Tool for Agricultural Research (STAR) software (http://bbi.irri.org/products) developed by IRRI’s Biometrics and Breeding Informatics team.

### Data availability statement

All data reported in this manuscript were obtained during this study and the data are included in the manuscript files as well as in the Supplementary information files.

### Ethical approval statement

All experiments conducted and reported in this manuscript were carried out following relevant guidelines and regulations of the government of the Philippines. The experiments conducted for this study were approved by the appropriate committee of the Institution.

## Electronic supplementary material

Supplementary Information

## References

[1] Seck PA, Diagne A, Mohanty S, Wopereis MCS (2012). Crops that feed the world 7: rice. Food Security.

[2] Khush GS (2005). What it will take to feed 5.0 billion rice consumers in 2030. Plant Mol. Biol..

[3] Ray DK, Mueller ND, West PC, Foley JA (2013). Yield trends are insufficient to double global crop production by 2050. PLoS One.

[4] Wang Y, Li J (2005). The plant architecture of rice (*Oryza sativa*). Plant Mol. Biol..

[5] Xing Y, Zhang Q (2010). Genetic and molecular bases of rice yield. Annu. Rev. Plant Biol..

[6] Miura K, Ashikari M, Matsuoka M (2011). The role of QTLs in the breeding of high-yielding rice. Trends Plant Sci..

[7] Huang R (2013). Genetic bases of rice grain shape: so many genes, so little known. Trends Plant Sci..

[8] Jena, K. K. & Nissila, E. A. J. Genetic improvement of rice (*Oryza sativa* L.). In: *Genetic improvement of tropical crops*. (Ed. Campos, H.) pp 111–127 (Springer, 2017).

[9] Jena, K. K. & Ramkumar, G. Achieving sustainable cultivation of rice. *Breeding strategies to improve rice yields: an overview*. (Ed. Sasaki, T.) pp 51–68 (Burleigh Dodds Science, 2017).

[10] Huang X (2009). Natural variation at the *DEP1* locus enhances grain yield in rice. Nat. Genet..

[11] Jiao Y (2010). Regulation of *OsSPL14* by OsmiR156 defines ideal plant architecture in rice. Nat. Genet..

[12] Xu Q (2014). Breeding value estimation of the application of *IPA1* and *DEP1* to improvement of *Oryza sativa* L. ssp. *japonica* in early hybrid generations. Mol. Breed..

[13] Ashikari M (2005). Cytokinin oxidase regulates rice grain production. Science.

[14] Ookawa T (2010). New approach for rice improvement using a pleiotropic QTL gene for lodging resistance and yield. Nat. Commun..

[15] Ohsumi A (2011). Evaluation of yield performance in rice near isogenic lines with increased spikelet number. Field Crops Res..

[16] Miura K (2010). *OsSPL14* promotes panicle branching and higher grain productivity in rice. Nat. Genet..

[17] Kakutani T (2002). Epi-alleles in plants: inheritance of epigenetic information over generations. Plant Cell Physiol..

[18] Zhang L (2017). A natural tandem array alleviates epigenetic repression of IPA1 and leads to superior yielding rice. Nat. Commun..

[19] Kim SR (2016). Development and validation of allele-specific SNP/indel markers for eight yield-enhancing genes using whole-genome sequencing strategy to increase yield potential of rice, *Oryza sativa* L. Rice.

[20] Peng S, Khush GS, Virk P, Tang Q, Zou Y (2008). Progress in ideotype breeding to increase rice yield potential. Field Crops Res..

[21] Dingkuhn M (2015). Improving yield potential of tropical rice: achieved levels and perspectives through improved ideotypes. Field Crops Res..

[22] Fujita D (2013). *NAL1* allele from a rice landrace greatly increases yield in modern indica cultivars. Proc. Natl. Acad. Sci. USA.

[23] Liao CY, Wu P, Hu B, Yi KK (2001). Effects of genetic background and environment on QTLs and epistasis for rice (*Oryza sativa* L.) panicle number. Theor. Appl. Genet..

[24] Barbary A (2014). The plant genetic background affects the efficiency of the pepper major nematode resistance genes Me1 and Me3. Theor. Appl. Genet.

[25] Doust AN (2014). Beyond the single gene: how epistasis and gene-by-environment effects influence crop domestication. Proc. Natl. Acad. Sci. USA.

[26] Zeng D (2017). Rational design of high-yield and superior-quality rice. Nat. Plants.

[27] Feng X (2017). Updating the elite rice variety Kongyu 131 by improving the Gn1alocus. Rice.

[28] Kobayashi A (1990). Breeding a new rice variety “Habataki”. *Bull*. *Hokuriku*. Natl. Agric. Exp. Stn. (Japan).

[29] Kim B (2014). Defining the genome structure of ‘Tongil’ rice, an important cultivar in the Korean “green revolution”. Rice.

[30] Khush, G. S. & Virk, P. S. IR varieties and their impact. International Rice Research Institute, Los Baños, Philippines. (IRRI, 2005).

[31] Lu Z (2013). Genome-wide binding analysis of the transcription activator IDEAL PLANT ARCHITECTURE1 reveals a complex network regulating rice plant architecture. Plant Cell.

[32] Wang J (2017). Tissue-specific ubiquitination by IPA1 INTERACTING PROTEIN1 modulates IPA1 protein levels to regulate plant architecture in rice. Plant Cell.

[33] Song X (2017). IPA1 functions as a downstream transcription factor repressed by D53 in strigolactone signaling in rice. Cell Res..

[34] Peng S (2000). Grain yield of rice cultivars and lines developed in Philippines since 1996. Crop Sci..

[35] Laza MRC, Peng S, Akita S, Saka H (2003). Contribution of biomass partitioning and translocation to grain yield under sub-optimum growing conditions in irrigated rice. Plant Prod. Sci..

[36] Duitama J (2015). Whole genome sequencing of elite rice cultivars as a comprehensive information resource for marker assisted selection. PLoS One.

[37] Peng, S., Khush, G. S. & Cassman, K. G. Evolution of the new plant ideotype for increased yield potential. *Proceedings of a Workshop on Rice Yield Potential in Favorable Environments*. (Ed. Cassman, K. G.) pp. 5–20 (IRRI, 1994)

[38] Yuan LP (1997). Super-high yield hybrid rice breeding. Hybrid Rice.

[39] Zhang GH, Xu Q, Zhu XD, Qian Q, Xue HW (2009). SHALLOT-LIKE1 is a KANADI transcription factor that modulates rice leaf rolling by regulating leaf abaxial cell development. Plant Cell.

[40] Wang B, Wang H (2017). *IPA1*: a new “Green Revolution” gene. Mol. Plant.

[41] Kim SR, Yang J, An G, Jena KK (2016). A simple DNA preparation method for high quality polymerase chain reaction in rice. Plant Breed. Biotechnol..

[42] Kim SR, Jeon JS, An G (2011). Development of an efficient inverse PCR method for isolating gene tags from T-DNA insertional mutants in rice. Methods Mol. Biol..

[43] Wolfe D, Dudek S, Ritchie MD, Pendergrass SA (2013). Visualizing genomic information across chromosomes with PhenoGram. BioData Mining.

