# Supplementary material for: Introgression of a functional epigenetic OsSPL14WFP allele into elite indica rice genomes greatly improved panicle traits and grain yield
Source: Sci Rep. 2018 Mar 1;8:3833. doi: 10.1038/s41598-018-21355-4 (PMC5832747; doi:10.1038/s41598-018-21355-4)
Supplement: Supplementary file 1 — Supplementary Information [file 41598_2018_21355_MOESM1_ESM.pdf]

Introgression of a functional epigenetic *OsSPL14*^{WFP} allele into elite indica rice genomes greatly improved panicle traits and grain yield

Sung-Ryul Kim[1], Joie M. Ramos[1], Rona Joy M. Hizon[1], Motoyuki Ashikari[2], Parminder S. Virk[3], Edgar A. Torres[4], Eero Nissila[1] and Kshirod K. Jena[1*]

[1] Strategic Innovation Platform, International Rice Research Institute (IRRI), DAPO Box 7777, Metro Manila, Philippines. [2] Bioscience and Biotechnology Center, Nagoya University, Nagoya, Japan. [3] International Center for Tropical Agriculture (CIAT), A.A. 6713, Cali, Colombia. [4] Rice Tech LTDA, Santa Maria, RS, Brazil.

[*]**Correspondence:**

Kshirod K. Jena

Tel: +63 2 580 5600; Email: k.jena@irri.org

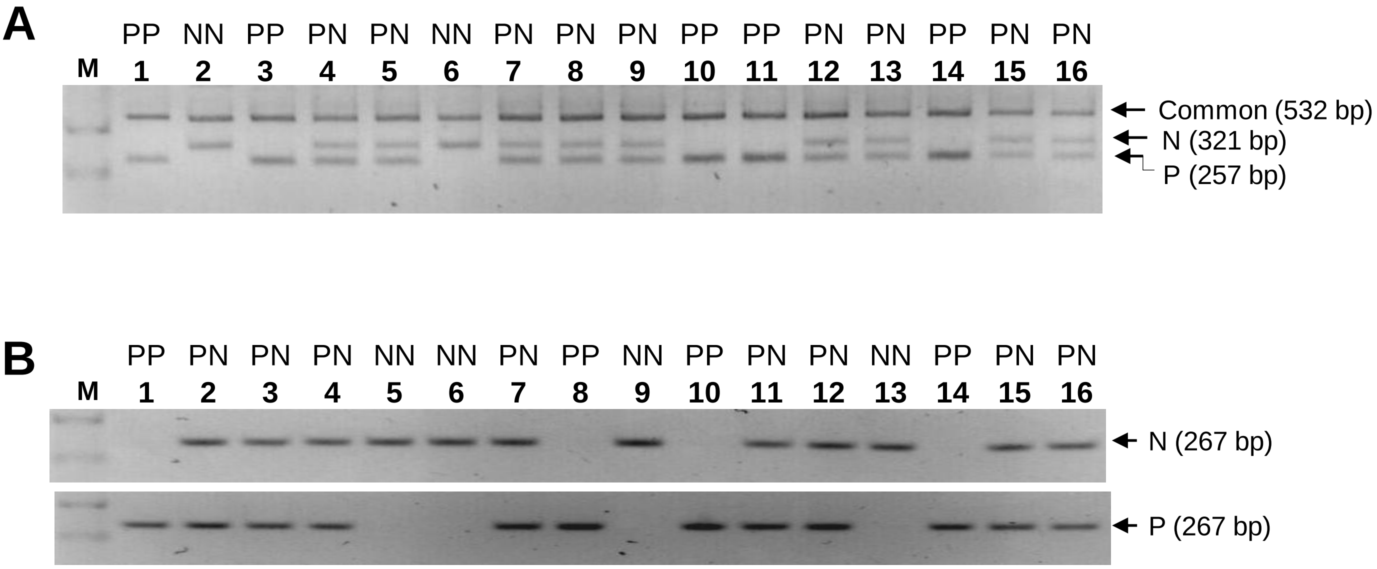

Supplementary Figure S1. Agarose gel images of the allele specific markers used in this study. The Gn1a-17 SNP marker (**A**) and the SPL14-04 SNP marker (**B**) was applied for genotyping of the lines YP15-766 and YP15-767, respectively. For examples, the genotype of 16 BC_3F_2 plants are presented. The plant number with genotype was shown on top of the gel image. N, non-target allele; P, yield-positive allele; M, DNA size marker.

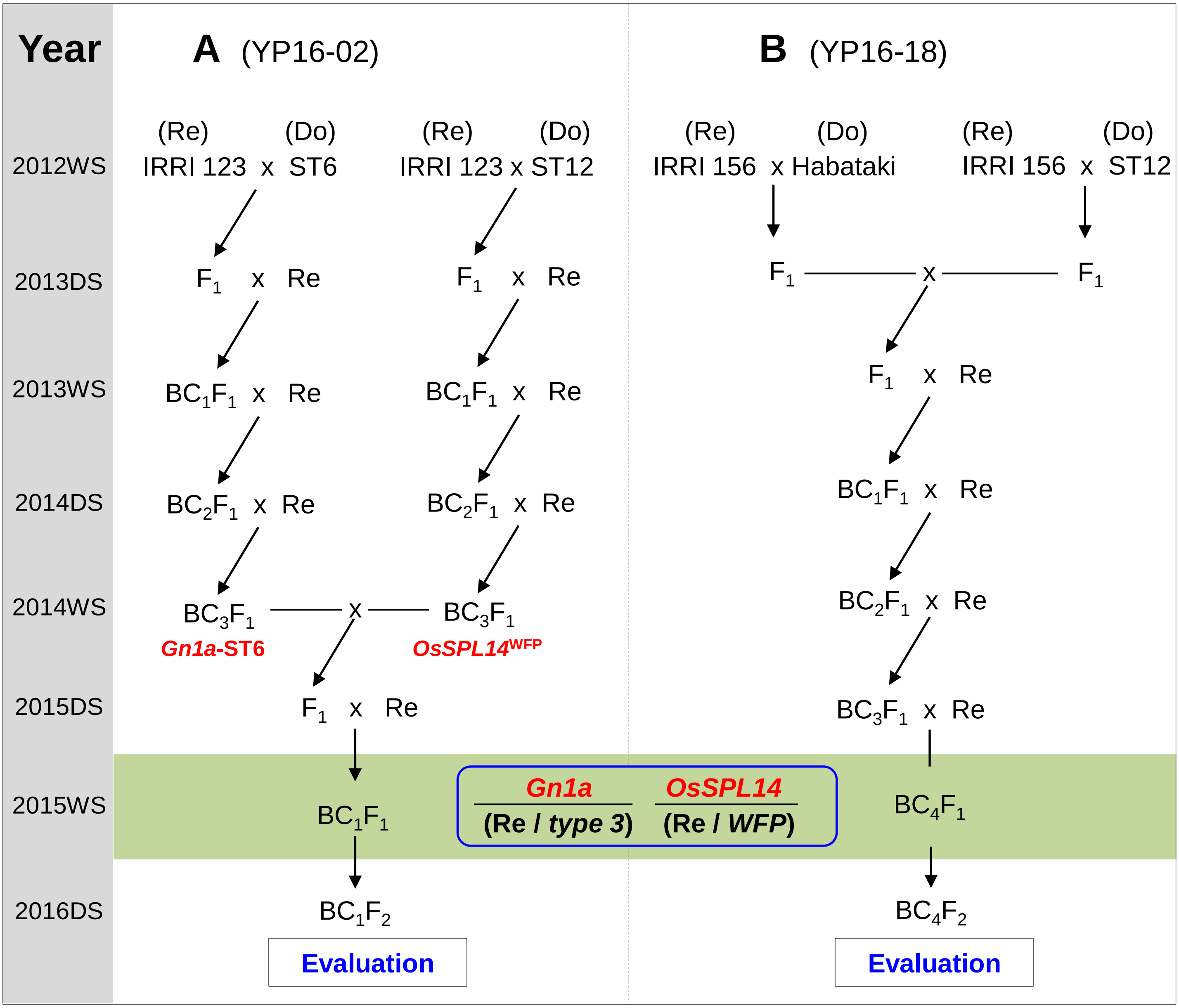

Supplementary Figure S2. Schematic representation of the line development procedures for combining *Gn1a*-type 3 and *OsSPL14*^{WFP} alleles. In every season, MAS was conducted with the Gn1a-17 SNP and SPL14-04 SNP markers. In 2015WS, a single heterozygous plant to both genes (highlighted by green background color) was self-pollinated to generate homozygous progenies. Re, recipient parent; Do, donor parent. (**A**) Line YP16-02. *Gn1a*-type 3 allele was derived from the donor ST6. (**B**) Line YP16-18. *Gn1a*-type 3 allele was derived from either Habataki or ST12.

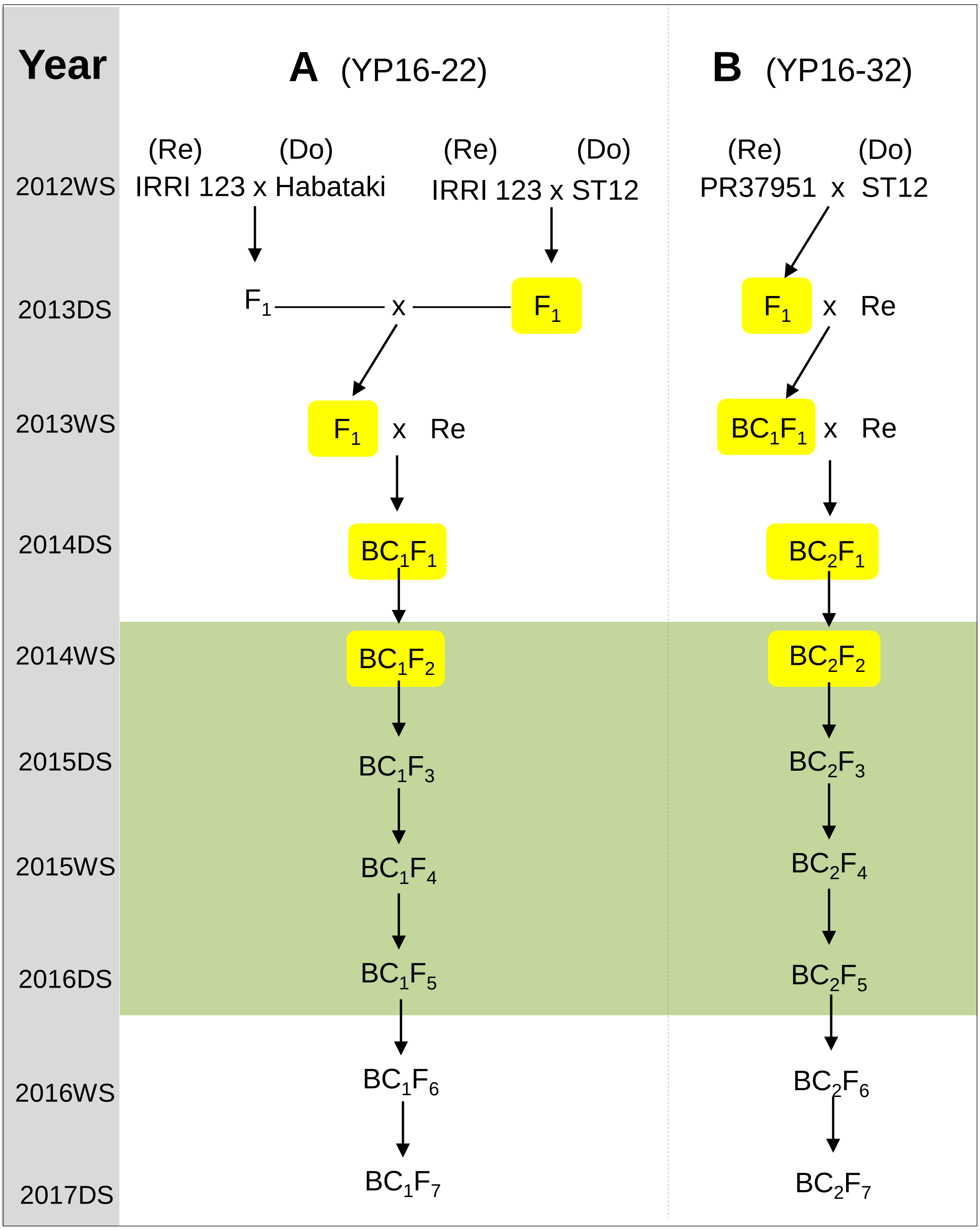
Year
A (YP16-22)
B (YP16-32)
2012WS
(Re) (Do)
IRRI 123 x Habataki
(Re) (Do)
IRRI 123 x ST12
(Re) (Do)
PR37951 x ST12
2013DS
F1 x F1
F1 x Re
2013WS
F1 x Re
BC1F1 x Re
2014DS
BC1F1
BC2F1
2014WS
BC1F2
BC2F2
2015DS
BC1F3
BC2F3
2015WS
BC1F4
BC2F4
2016DS
BC1F5
BC2F5
2016WS
BC1F6
BC2F6
2017DS
BC1F7
BC2F7

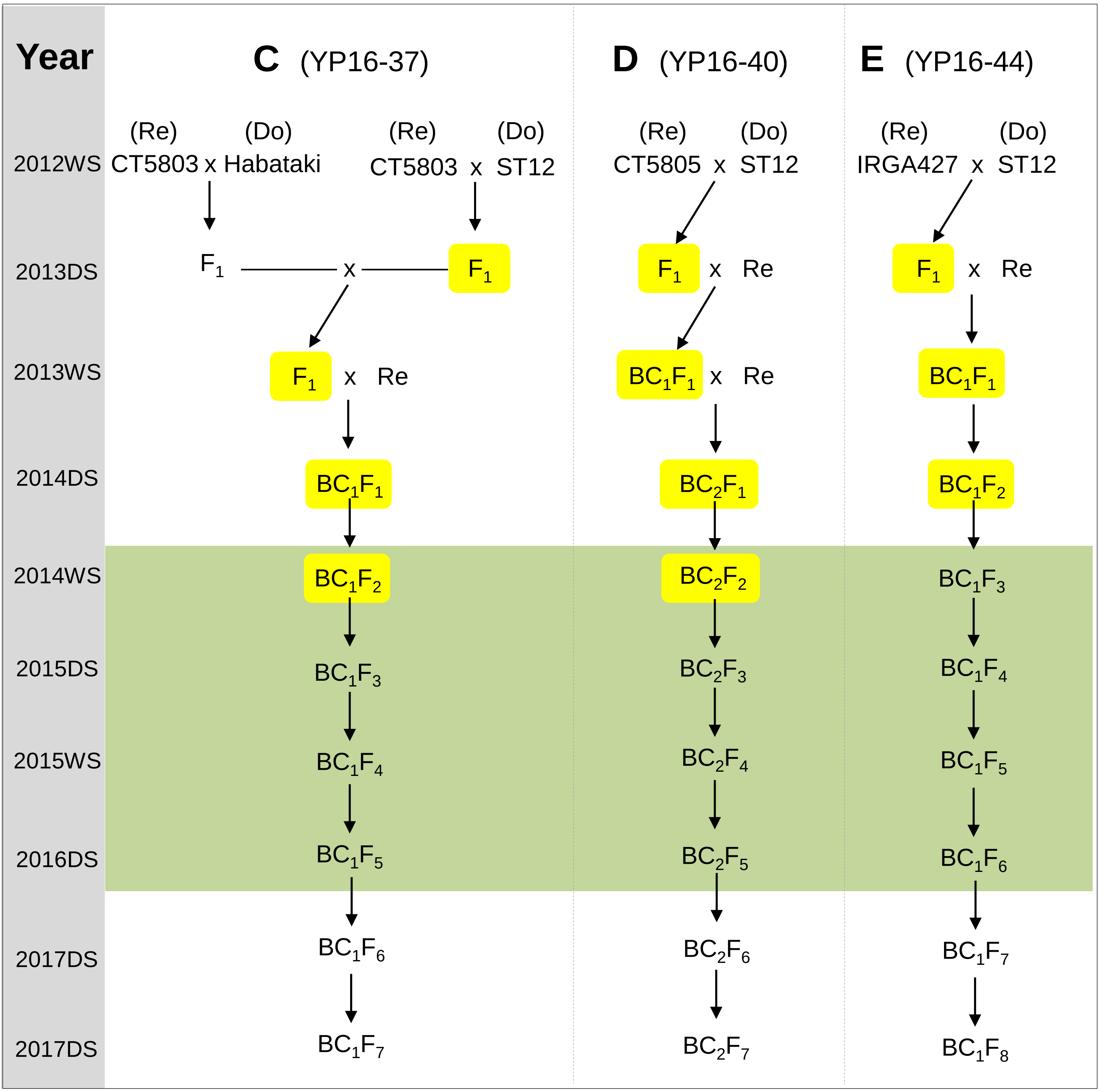

Supplementary Figure S3. Schematic representation of the combined breeding approaches (MAS with plant selection) for breeding of high-yielding lines. Intermediate breeding lines highlighted by yellow box were genotyped by the SPL14-04 SNP marker. Plant selections were conducted for four cropping seasons (2014WS-2016DS, highlighted by green background color) in the field. Re, recipient parent; Do, donor parent. (**A**) Line YP16-22. (**B**) Line YP16-32. (**C**) Line YP16-37. (**D**) Line YP16-40. (**E**) Line YP16-44.

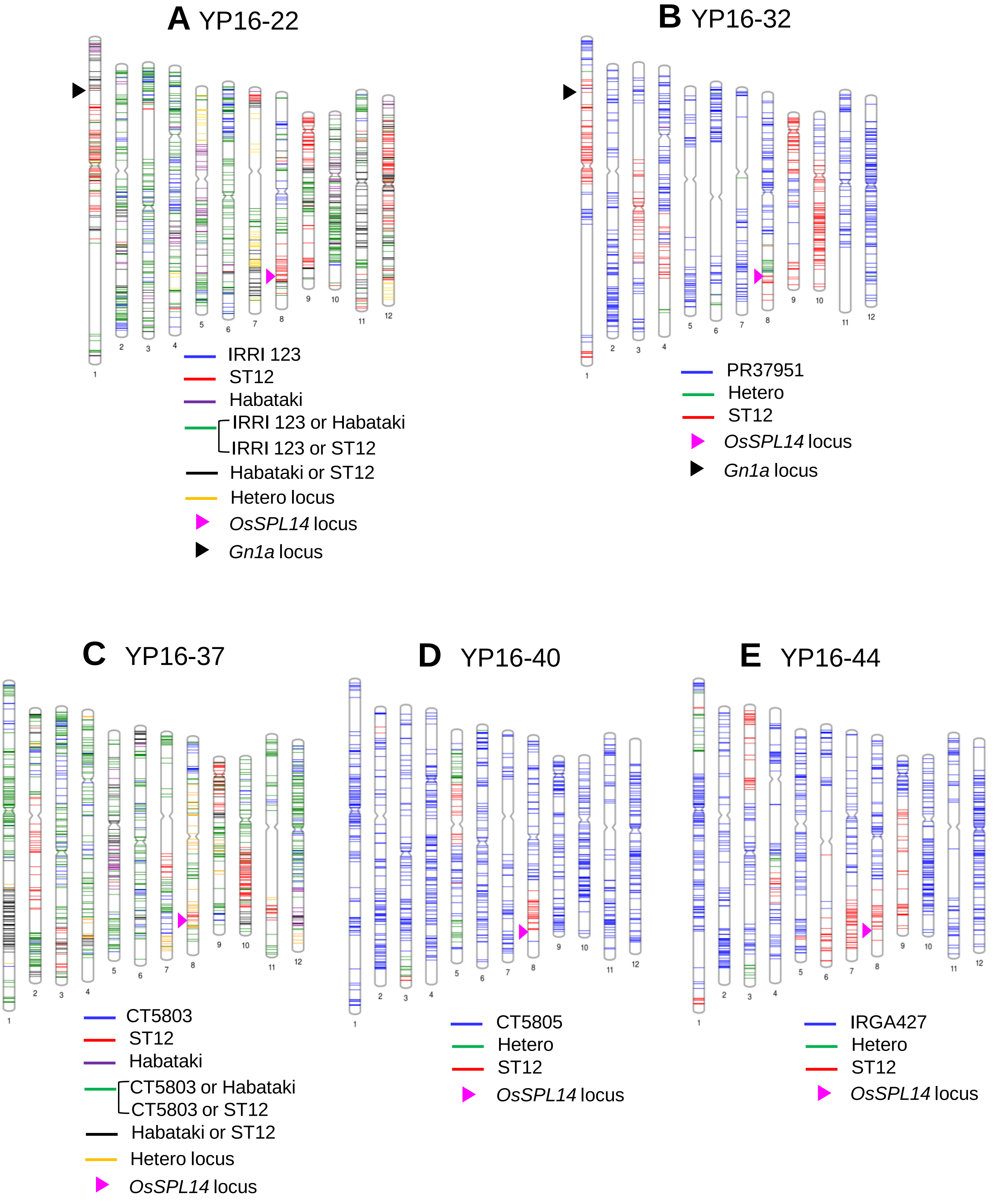

Supplementary Figure S4. Graphical genotype maps of the five selected high-yielding lines. (**A**) Line YP16-22. (**B**) Line YP16-32. (**C**) Line YP16-37. (**D**) Line YP16-40. (**E**) Line YP16-44.

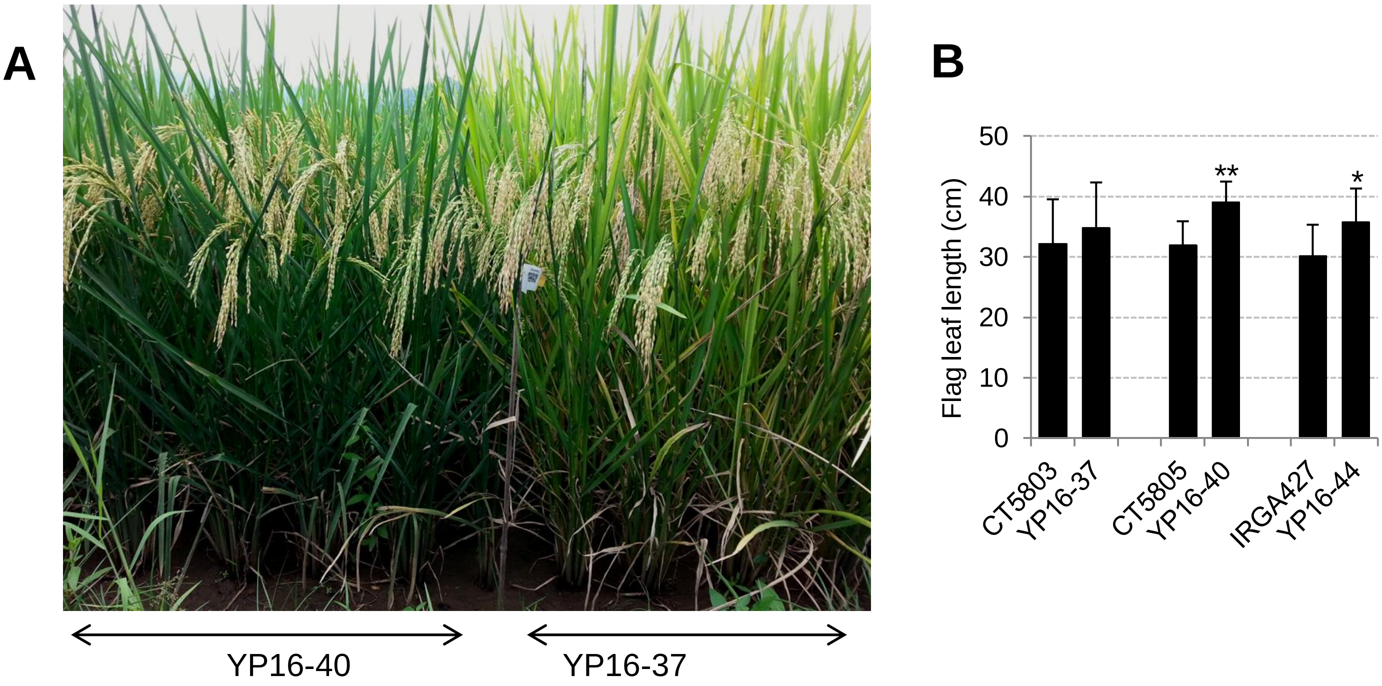

Supplementary Figure S5. Phenotypes of line YP16-40. (**A**) Phenotype in the field. (**B**) Comparison of flag leaf length between the selected line and its background variety.

Supplementary Table S1. Analysis of genetic segregation pattern of *Gn1a* and *OsSPL14* genes in BC_3F_2 populations using the chi-squre (χ^2) test

Line no.		*Gn1a* allele				
		Recipient	Hetero	Donor	Total	χ^2
YP15-766	Observed number (O)	12	27	9	48	
	Expeted number (E)	12	24	12	48	
	$(O-E)^2/E$	0.000	0.375	0.750		1.125
YP15-752	Observed number (O)	9	25	12	46	
	Expeted number (E)	11.5	23	11.5	46	
	$(O-E)^2/E$	0.543	0.174	0.022		0.739
YP15-774	Observed number (O)	10	15	7	32	
	Expeted number (E)	8	16	8	32	
	$(O-E)^2/E$	0.500	0.063	0.125		0.688
YP15-775	Observed number (O)	8	15	9	32	
	Expeted number (E)	8	16	8	32	
	$(O-E)^2/E$	0.000	0.063	0.125		0.188
		OsSPL14 allele				
		Recipient	Hetero	Donor	Total	χ^2
YP15-767	Observed number (O)	11	28	11	50	
	Expeted number (E)	12.5	25	12.5	50	
	$(O-E)^2/E$	0.180	0.360	0.180		0.720
YP15-746	Observed number (O)	8	23	14	45	
	Expeted number (E)	11.25	22.5	11.25	45	
	$(O-E)^2/E$	0.939	0.011	0.672		1.622
YP15-754	Observed number (O)	17	24	7	48	
	Expeted number (E)	12	24	12	48	
	$(O-E)^2/E$	2.083	0.000	2.083		4.167
YP15-786	Observed number (O)	12	23	13	48	
	Expeted number (E)	12	24	12	48	
	$(O-E)^2/E$	0.000	0.042	0.083		0.125

The expected number was set based on the normal Mendelian segregation pattern of the single gene (homozygous for recipient allele: heterozygous: homozygous for donor allele = 1:2:1 ratio).

Supplementary Table S2. Genome recovery rate of the selected BC_3F_2 plants from eight different populations

Target allele	Lin No.	Recipient	Donor	No. of polymorphic SNP[a]	No. of SNP allele			Genome ratio (%)[b]	
					Recipient	Donor	Hetero	Recipient	Donor
Gn1a-type 3									
	YP15-766	PR37951	Habataki	760	594	44	122	86.2	13.8
	YP15-752	IRRI 146	ST12	899	766	39	94	90.4	9.6
	YP15-774	CT5803	Habataki	635	607	8	20	97.2	2.8
	YP15-775	CT5803	ST6	1,657	1,411	15	231	92.1	7.9
OsSPL14^{WFP}									
	YP15-767	PR37951	ST12	916	800	27	89	92.2	7.8
	YP15-746	IRRI 123	ST12	909	700	75	134	84.4	15.6
	YP15-754	IRRI 154	ST12	918	788	114	16	86.7	13.3
	YP15-786	CT5805	ST12	1,082	976	33	73	93.6	6.4

[a] Total polymorphic SNPs between bi-parents among ~6,000 SNP markers.
[b] Genome ratio was calculated based on the number of SNP markers.

Supplementary Table S3. Agronomic traits of the BC_3F_2 populations for evaluating *Gn1a*-type 3 and *OsSPL14*^{WFP} alleles

Line	Gene-allele	PH (cm)	TN	PL (cm)	PBN	SBN	GNPP	SF (%)
YP15-766	*Gn1a*-PR37951	107.3	14	27.9	11.8	54.1	227.8	78.8
YP15-766	*Gn1a*-Habataki	106.9	11.4	28.3	11	46.7	205.1	86.9
YP15-752	*Gn1a*-IRRI 146	87	18.4	23.6	11.4	28	163	78.3
YP15-752	*Gn1a*-ST12	84	17.5	22.9	12	28.1	163.9	73.6
YP15-774	*Gn1a*-CT5803	98.6	12.3	28.2	9.1	26.5	157.9	86.9
YP15-774	*Gn1a*-Habataki	101.2	14.6	28.2	9.8*	26.9	162.8	86.6
YP15-775	*Gn1a*-CT5803	100.3	13.8	28	9.5	33.2	175.8	85.1
YP15-775	*Gn1a*-ST6	98.6	12.3	27.5	10.4	37.7	200.1	87.5
YP15-767	*OsSPL14*-PR37951	105.8	13.1	28.2	11.8	45.5	187.4	73
YP15-767	*OsSPL14*-ST12	105	12.9	28.2	16.1**	72.8**	288.5**	70.3
YP15-746	*OsSPL14*-IRRI 123	90	17	25	10.2	28.1	131.5	90.7
YP15-746	*OsSPL14*-ST12	93	10.8*	23.6	14.3*	38.5*	206.6*	82.6
YP15-754	*OsSPL14*-IRRI 154	100.4	12.9	23.3	11.5	37.4	151.9	81.6
YP15-754	*OsSPL14*-ST12	104.2	10.8	23.2	19.3**	53.3*	242.0**	75.9
YP15-786	*OsSPL14*-CT5805	109.1	10.4	29.4	13	58.8	238.2	84.7
YP15-786	*OsSPL14*-ST12	112.4	7.9	30.8	20.7**	71.6*	313.7**	75.5*
PR37951		98.6	12.4	27.6	11.6	47.4	200.4	84.2
IRRI 123		104.1	20.6	22.6	10.2	34.8	164.8	91.9
IRRI 146		84.2	18.8	22.9	11.2	45	184.2	90.8
IRRI 154		98.3	13.8	23.8	11.8	61.4	242.6	92
CT5803		100.6	16.8	27.6	9.6	38.8	170	91.9
CT5805		112.2	14.6	30.2	14.2	83.8	324.8	89.4

Asterisks represent significant difference between two alleles based on Student's *t*-test (* $\alpha = 0.05$ and ** $\alpha = 0.01$). (n = 5 to 9 plants) PH: plant height, TN: tiller number, PL: panicle length, PBN: primary branching number of panicle, SBN: secondary branching number of panicle, GNPP: grain number per panicle, SF: spikelet fertility

Supplementary Table S4. Agronomic traits of the BC_3F_3 populations for evaluating *Gn1a*-type 3 and *OsSPL14*^{WFP} alleles

Line	Gene-allele	DTH	PH (cm)	TN	PL (cm)	PBN	SBN	GNPP	SF (%)	TGW (g)
YP15-766	*Gn1a*-PR37951	85.7	119.6	12.5	30.6	12.6	50.8	204	74.5	24.4
YP15-766	*Gn1a*-Habataki	85.3	123.5	13.4	31.1	12.3	52.6	207.1	71	24.5
YP15-752	*Gn1a*-IRRI 146	97	101.8	17.3	23.9	13.4	34.5	170	72.9	19.5
YP15-752	*Gn1a*-ST12	96	103.1	21.2**	25.0**	13	36.4	166.8	74.9	19.4
YP15-774	*Gn1a*-CT5803	93.3	113.6	13.1	31.1	12.4	45.3	187.6	72.4	26
YP15-774	*Gn1a*-Habataki	92	116.5	12.3	31.4	12.1	45	195.3	68.6	26.2
YP15-767	*OsSPL14*-PR37951	86.3	104.9	13.1	28.1	14.5	63.1	249.8	64.5	21.7
YP15-767	*OsSPL14*-ST12	85.3	115.3*	10.9*	29.8**	19.4**	77.3**	305.5**	68	22.8*
YP15-746	*OsSPL14*-IRRI 123	91.5	111	15.6	25.9	12.6	33.7	134.2	82.4	23.5
YP15-746	*OsSPL14*-ST12	92.3	114.1	12.9*	25.8	19.4**	39.5**	186.5**	76.6*	25.6**
YP15-754	*OsSPL14*-IRRI 154	99	127.7	11.9	25.2	16.2	56.3	224.6	58.3	24.5
YP15-754	*OsSPL14*-ST12	97.3	127.4	11.1	25.8*	18	66.6*	248.3*	62.1	24.5
PR37951		85	116.2	13.6	30.2	12.2	49.7	202.2	74.1	24.6
IRRI 146		94	98.4	19	21.7	12	26.6	121.9	73.2	21.2
CT5803		92	113.4	12.2	29.9	11.5	44.5	173.7	79.3	25.9
IRRI 123		96	111	16.2	23.3	11.8	23.3	105.7	84.9	27.1
IRRI 154		95	120.4	14.2	27.7	12.8	52.9	251.1	63.7	25.3

Asterisks represent significant difference between two allele based on Student's *t*-test (* $\alpha = 0.05$ and ** $\alpha = 0.01$). ($n = 15$ plants). DTH: days to heading, PH: plant height, TN: tiller number, PL: panicle length, PBN: primary branching number of panicle, SBN: secondary branching number of panicle, GNPP: grain number per panicle, SF: spikelet fertility, TGW: 1,000-grain weight

Supplementary Table S5. Analysis of the allele segregation pattern in the two genes combined lines using chi-square test

Line		Genotype[a]									Total	χ2
		AABB	*AAbb*	*aaBB*	*aabb*	*AABb*	*AaBB*	*Aabb*	*aaBb*	*AaBa*		
	Expected segregation ratio[b]	1	1	1	1	2	2	2	2	4	16	
YP16-02	Observed number (O)	16	10	16	15	24	32	32	28	61	234	
	Expeted number (E)	14.625	14.625	14.625	14.625	29.250	29.250	29.250	29.250	58.500	234	
	$(O-E)^2/E$	0.129	1.463	0.129	0.010	0.942	0.259	0.259	0.053	0.107	3.350	3.350
YP16-18	Observed number (O)	10	10	11	11	16	32	24	22	56	192	
	Expeted number (E)	12	12	12	12	24	24	24	24	48	192	
	$(O-E)^2/E$	0.333	0.333	0.083	0.083	2.667	2.667	0.000	0.167	1.333	7.667	7.670

[a] *A* = *Gn1a* donor allele, *a* = *Gn1a* recipient allele, *B* = *OsSPL14* donor allele, *b* = *OsSPL14* recipient allele
[b]Expected segregation ratio was set based on the normal Mendelian segregation pattern of the two independent genes.

Supplementary Table S6. Yield and agronomic traits of the selected high-yielding lines in 2016DS

Plant materials[a]	DTH	PH (cm)	TN	PL (cm)	PBN	SBN	GNPP	SF (%)	TGW (g)	Grain yield[b] (t/ha)	Yield (%) to RP[c]
IRRI 123	81.3	100.9	15.1	23.7	10.3	29.9	124.7	89.7	23.2	7.01±0.32	
YP16-22	67.3*	96.9	11.3	25.3	15.3**	90.4**	402.2**	85.8	21.6	7.57±0.44	108.0
PR37921	85.7	106.6	14.2	28.7	11.1	49.4	200.2	90.0	24.2	Not tested	
YP16-32	84.7	114.5*	12.1	27.0	16.0**	89.1**	368.9**	81.3	20.1	8.06±0.74	Not tested
CT 5803	86.3	106.1	15.9	29.2	10.7	43.0	173.4	87.7	26.6	8.83±0.92	
YP16-37	80.0	121.3**	12.7	30.4	12.9*	61.7**	257.2**	95.0	28.9**	10.05±1.35	113.8
CT 5805	91.3	115.2	12.7	31.0	13.4	67.2	226.4	81.3	27.2	7.47±1.40	
YP16-40	90.7	120.8*	10.1	32.3	22.9**	121.0**	435.1**	85.3	24.2	8.91±1.91	119.3
IRGA427	89.3	95.3	16.3	21.8	9.8	37.9	151.7	85.0	23.3	5.13±0.71	
YP16-44	96.7*	103.7**	12.5	24.0*	18.2**	82.1**	363.0**	80.5	24.9	6.98±1.39	136.0

[a] Five high-yielding lines YP16-22, YP16-32, YP16-37, YP16-40, and YP16-44 were compared with their background cultivars IRRI 123, PR37921, CT5803, CT5805, and IRGA427, respectively.

[b]Average grain yield of 5 m^2 plot from the three replications was converted to tons per hectare (t/ha).

[c]Percentage yield compared to each recurrent parent (RP)

Significant difference between the breeding line and its recurrent parent was calculated based on Student's *t*-test (* $\alpha = 0.05$ and ** $\alpha = 0.01$). DTH: days to heading, PH: plant height, TN: tiller number, PL: panicle length, PBN: primary branching number of panicle, SBN: secondary branching number of panicle, GNPP: grain number per panicle, SF: spikelet fertility, TGW: 1,000-grain weight
